# Memristive Physical Reservoir Computing

**DOI:** 10.1002/advs.75476

**Published:** 2026-04-28

**Authors:** Dian Jiao, Ziyuan Wang, Jingrui Wang, Li Zhang, Xianhua Wei, Lingxiang Hu, Zongxiao Li, Athanasios V. Vasilakos, Zhizhen Ye, Fei Zhuge

**Affiliations:** ^1^ School of Materials Science and Engineering Shanghai University Shanghai China; ^2^ Ningbo Institute of Materials Technology and Engineering Chinese Academy of Sciences Ningbo China; ^3^ Ningbo Key Laboratory of Optoelectronic Information Perception Technology, School of Electronic and Information Engineering Ningbo University of Technology Ningbo China; ^4^ Healthcare Engineering Centre School of Engineering Temasek Polytechnic Singapore Singapore; ^5^ State Key Laboratory of Environment‐friendly Energy Materials Southwest University of Science and Technology Mianyang China; ^6^ Center for AI Research University of Agder Grimstad Norway; ^7^ State Key Laboratory of Silicon and Advanced Semiconductor Materials School of Materials Science and Engineering Zhejiang University Hangzhou China; ^8^ Institute of Wenzhou Zhejiang University Wenzhou China; ^9^ Center for Excellence in Brain Science and Intelligence Technology Chinese Academy of Sciences Shanghai China

**Keywords:** brain‐inspired neuromorphic computing, memristors, optoelectronic memristors, physical reservoir computing, reservoir computing

## Abstract

Reservoir computing (RC) has emerged as an efficient neuromorphic framework for temporal information processing, offering low training complexity and hardware‐friendly implementation. Memristors’ nonlinear dynamics and input‐dependent memory effects make them ideal candidates for high‐performance physical RC. Based on their conductance modulation, memristors can be classified as electronic or optoelectronic types. However, no systematic review has compared electrically and optically controlled memristive RC. This review fills that gap by comparing them from the device to the system level. We first summarize the resistive switching mechanisms of electronic and optoelectronic memristors, highlighting their distinct roles in RC encoding and processing temporal signals. We then review recent advances in electronic memristive RC, emphasizing architecture innovations and performance improvements in pattern recognition and sequence prediction. Subsequently, we focus on optoelectronic memristive RC, where the high parallelism of optical inputs are harnessed for color vision processing, dynamic gesture recognition, and multi‐signals fusion. Notably, we provide a systematic comparison between single‐modal and multi‐modal RC implementations, demonstrating how hybrid electro‐optical stimulation enhances feature diversity and task accuracy. Finally, we outline key challenges and future research directions, including the development of fully hardware‐integrated RC systems, system‐level multi‐modal RC architectures, and novel encoding paradigms.

## Graphical abstract

Memristors’ nonlinear dynamics and input‐dependent memory effects make them ideal candidates for high‐performance physical reservoir computing (RC). Based on their conductance modulation, memristors can be classified as electronic or optoelectronic types. However, no systematic review has compared electrically and optically controlled memristive RC. This review fills that gap by comparing them from the device to the system level.

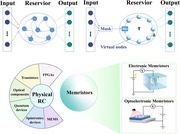

## 1 Introduction

The wave of artificial intelligence (AI) is becoming the central driving force behind contemporary industrial transformation and technological innovation [1]. AI methodologies have been widely deployed across diverse fields, including healthcare [2, 3, 4], autonomous driving [5, 6, 7], environmental science [8, 9], astronomical exploration [10], and financial analysis [11, 12], where the ability to process large volumes of complex data efficiently is essential. At the core of these advances lies the continuous development of artificial neural networks (ANNs), which form the computational backbone of modern AI systems [13].

Among ANN architectures, convolutional neural networks (CNNs) have achieved remarkable success in processing grid‐like data, particularly in image recognition task [14]. By leveraging local connectivity and hierarchical feature extraction through convolutional layers, CNNs efficiently extract spatial correlations from input data. However, their inherent feedforward architecture limits their ability to process temporal information. To address this challenge, recurrent neural networks (RNNs) incorporate recurrent connections within hidden layers, enabling the modeling of temporal dependencies and nonlinear dynamic behaviors [15, 16, 17]. Despite their powerful capability, conventional RNNs suffer from significant drawbacks, including complex network structures [18, 19, 20], heavy computational demands [20, 21], and gradient explosions [22, 23, 24]. These limitations pose major obstacles to scalable deployment, particularly in real‐time and hardware‐constrained applications.

**FIGURE 1:**
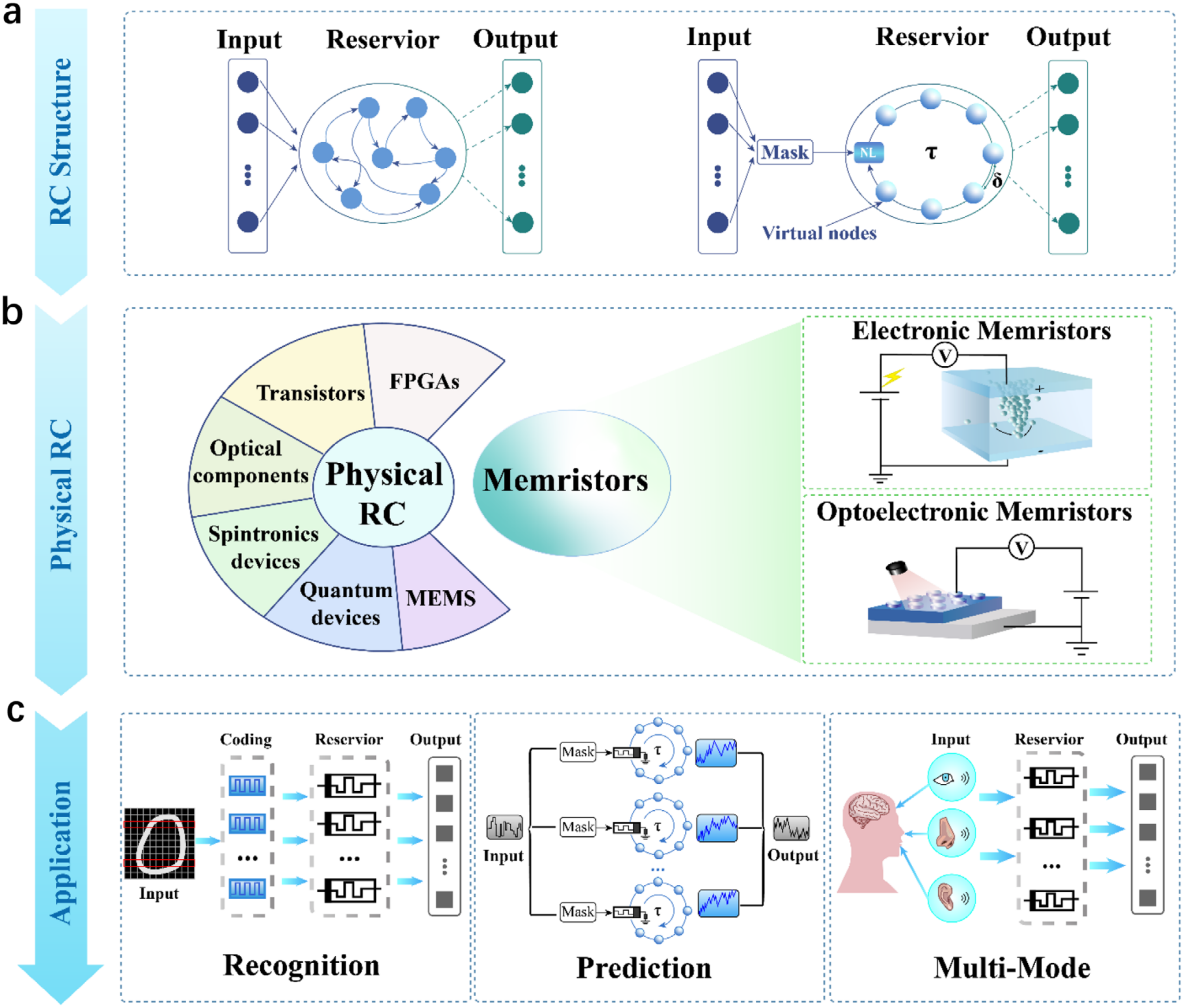
(a) Schematic of the classic RC and delay RC structures, comprising the input layer, reservoir, and readout layer. (b) Hardware implementations of physical reservoir computing. The two device types shown on the right are typical memristor configurations employed in physical RC systems. (c) The application domains of RC currently include recognition, prediction, and multimodal tasks.

Reservoir computing (RC) has emerged as an attractive alternative framework that effectively addresses many of these challenges [25, 26, 27, 28, 29, 30, 31]. As a modified computational framework for RNNs, RC replaces the trainable recurrent layers with a fixed nonlinear reservoir. The reservoir acts as a high‐dimensional dynamical system that nonlinearly maps input signals into rich state representations, making RC highly efficient for temporal signal processing. Since an RC system only requires training the weights of the output layer while the internal connections of the reservoir are randomly generated and remain fixed, the need for the complex gradient descent training process in conventional RNNs is eliminated. This significantly reduces training complexity and computational cost. Moreover, by omitting the recurrent connections between individual nodes present in traditional RNNs, RC systems can substantially decrease the number of components and circuit complexity in hardware implementations. The left panel of Figure 1a illustrates, a classical RC system comprises three key components: an input layer, a reservoir and a readout network [17, 20, 32, 33, 34].

Further extending the RC paradigm, Appeltant et al. enhanced the traditional RC system by introducing a delay RC scheme [35], as shown in right panel of Figure 1a. Delay RC utilizes nonlinear nodes in conjunction with a delay feedback loop to generate multiple virtual nodes through time multiplexing [26, 36, 37]. The linear weighted sum of these virtual node states forms the RC output. Delay RC systems exhibit rich dynamic characteristics for time series processing tasks. Whether using traditional RC or delay RC, temporal signals are processed within a fixed reservoir, making it more suitable for classification and prediction tasks involving time‐related information.

While software‐based RC implementations reply on numerical simulations of reservoir dynamics using complex mathematical models [38, 39, 40], such approaches are often computational inefficient and energy intensive, limiting their practicality for real‐time performance and energy efficiency [41]. Physical RC overcomes these limitations by directly exploiting the intrinsic complex responses of physical systems—such as nonlinear devices or optical components—as reservoirs [23, 42]. In this paradigm, the dynamic responses of physical systems to input stimuli naturally perform high‐speed and low‐power computation [43, 44, 45, 46]. In principle, any physical system exhibiting rich dynamic and fading memory can serve as a reservoir [20]. Consequently, a wide range of physical platforms, including field‐programmable gate arrays (FPGAs) [47, 48, 49, 50], micro‐electro‐mechanical systems (MEMS) [51, 52, 53], transistors [54, 55, 56, 57, 58, 59, 60, 61], memristors [41, 43, 62, 63, 64, 65, 66], quantum devices [67, 68, 69, 70], spintronics devices [71, 72, 73] and optical components [74, 75, 76], have been explored for hardware RC implementations (Figure 1b).

Among these platforms, memristors have attracted particular interest due to their intrinsic suitability for RC [31, 77]. This suitability arises from their ability to directly fulfill the core requirements of a physical reservoir. First, the resistance values of memristors depend on the history of current excitation, exhibiting a relaxation behavior known as fading memory. This property is essential for RC, as it provides the necessary short‐term memory to encode and retain temporal information from sequential inputs [78, 79, 80, 81]. Second, memristors possess an inherent nonlinear electrical response. This nonlinearity is vital because it enables the reservoir to perform a high‐dimensional, nonlinear transformation of input signals, thereby projecting them into a rich state space that enhances feature separability for the subsequent readout layer [82, 83, 84]. Third, memristors can be integrated into high‐density crossbar arrays, allowing efficient in‐memory matrix multiplication and accelerating the training of the readout layer [41, 63, 85, 86, 87, 88, 89, 90]. These features collectively position memristors as a highly promising hardware platform for scalable and energy‐efficient RC systems.

Beyond purely electrically controlled memristors, optoelectronic RC systems—represented by optically controlled memristors—have recently emerged as a powerful hybrid approach that combines the advantages of both electronic and photonic technologies [91, 92, 93, 94]. Optical inputs offer benefits such as high bandwidth, low crosstalk, and parallel processing, while electrical outputs maintain full compatibility with standard CMOS integrated circuits. Furthermore, optoelectronic RC can be effectively incorporated into in‐sensor computing architectures, merging sensing and computational functions. This allows early‐stage signal processing at the sensor level, reducing data transmission, latency, and overall power consumption. Recent studies have demonstrated significant progress in optoelectronic memristor‐based RC technology for applications such as multimodal recognition and chaotic system forecasting (Figure 1c) [95, 96, 97].

In this review, we provide a comprehensive overview of the recent research advances in memristor‐based RC systems. We first introduce the fundamental operating principles and switching mechanisms of electronic memristors and optoelectronic memristors. We then systematically summarize the memristive RC architectural features, highlight their performance characteristics and representative applications. Finally, we discuss the current key challenges and outline future development directions for memristor‐enabled RC technologies.

## 2 Memristor Fundamentals

The memristor is recognized as the fourth fundamental passive circuit element, complementing the resistor, capacitor, and inductor. Its resistance exhibits nonlinear variation with applied current or voltage, a concept first proposed by Chua in 1971 [98]. The practical significance of this concept was established in 2008 [99], when HP Lab demonstrated that resistive random‐access memory (RRAM) devices exhibit memristive behavior. Beyond RRAMs [100, 101, 102], the memristor family encompasses a broad range of emerging memory technologies, including phase‐change memories [103, 104, 105, 106, 107], ferroelectric memories [73, 108], and magnetic memories [109, 110, 111]. Owing to their cross‐array architecture, memristors enable high‐density integration at the nanoscale, providing a pathway to extend Moore's Law and realizing large‐scale neuromorphic hardware [90, 112].

Nonlinearity is one of the key characteristics of memristors, reflecting their dynamic response capability to external stimuli [84, 113]. Typically, increased nonlinearity results in more complex dynamic behavior of the device, contributing to effective projection of input signals into a high‐dimensional feature space—an essential requirement for reservoir computing [114]. Moreover, memristor states depend on both current and historical inputs, including electrical or optical signals, exhibiting relaxation behavior across different time scales [26, 62, 115]. The memristor can demonstrate short‐term plasticity and long‐term plasticity depending on the duration of the relaxation time [43, 116]. Short‐term plasticity is characterized by rapid conductance decay following stimulation, whereas long‐term plasticity correspnds to persistent conductance modulation. The coexistence of short‐term and long‐term plasticity within a single device provides a physical platform for emulating temporal information processing, rendering memristors particularly well suited for RC implementations [117, 118, 119, 120, 121, 122].

The nonlinear dynamics and memory effects of memristors arise from their underlying microscopic resistive switching mechanisms [123, 124]. A detailed understanding of these mechanisms is essential for the precise regulation of device dynamics, facilitating the exploration of memristive computing systems that operate at more suitable timescales. Based on the principles of resistive state control, memristors can be categorized into electronic memristors and optoelectronic memristors. This section primarily discusses the working mechanisms of both electronic and optoelectronic memristors.

### 2.1 Electronic Memristors

Electronic memristors implement nonlinear resistance changes through electrical signals. By adjusting the direction and magnitude of the applied voltage, the device's resistance can be modulated accordingly. The changed resistance values also exhibit a certain degree of non‐volatility. This capability allows the same device to perform both information storage and computation [21, 77, 125, 126, 127, 128]. Memristors typically feature a straightforward sandwich structure, with a functional memristive material layer positioned between top and bottom electrodes [19, 41, 48, 129, 130]. The top electrode is usually a conductive metal, while the bottom electrode may be a metal or a heavily doped n‐type or p‐type semiconductor. Although these memristors depend on electrical signals to adjust resistance, they demonstrate distinct resistive switching mechanisms.

#### 2.1.1 Ion Migration

The ion migration model represents the most extensively studied resistive switching mechanism and is broadly classified into oxygen ion migration and metal ion migration. Within this framework, the conductive filament model stands as a widely recognized theory of resistive switching, and its formation has been experimentally observed directly [131]. The application of a high operating voltage induces the formation of a conductive filament within the active layer, bridging the top and bottom electrodes and switching the device from a high‐resistance state (HRS) to a low‐resistance state (LRS)—a process known as SET. Conversely, applying a reverse voltage to the device in the LRS generates significant Joule heating, which ruptures the filament and returns the device to the HRS, completing the RESET process. Essentially, the composition of the conductive filament depends on the material system of the memristive devices [132].

**FIGURE 2:**
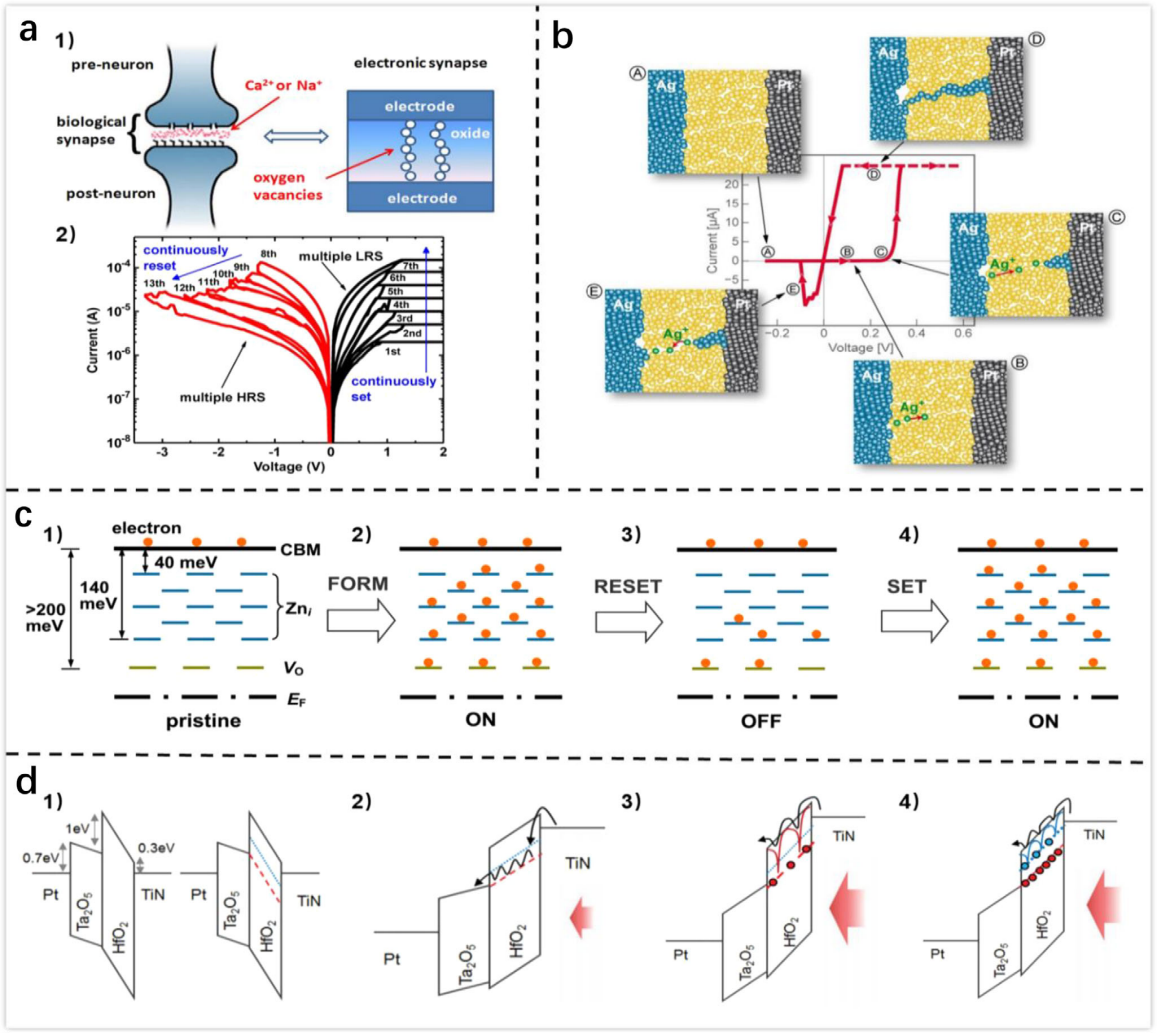
Mechanism of resistive switching. (a) Oxygen vacancy and b) Ag^+^ migration for conductive filament formation. Reproduced with permission [143]. Copyright 2011, IEEE (a). Reproduced with permission [145]. Copyright 2011, IOP Publishing (b). (c) The SCLC resistive switching scheme utilizing bulk conduction properties enabled by material defects. Reproduced with permission [160]. Copyright 2016, AIP Publishing. (d) Schematic illustration of resistive switching based on the Poole‐Frenkel mechanism. Reproduced with permission [164]. Copyright 2014, Wiley‐VCH.

In metal oxide‐based memristors (e.g., HfO_2_ [133, 134, 135], TiO_2_ [136, 137, 138], NiO [139, 140]), the filaments typically consist of oxygen vacancies. Under an electric field, oxygen ions migrate toward the anode, leaving behind a high density of oxygen vacancies that aggregate along the field direction to eventually form a conductive path [141, 142]. For example, in 2011, Yu and colleagues developed a bilayer HfO_x_/AlO_x_ memristive synapse [143]. Its conductance changes under external electrical stimulation as oxygen ions migrate, resembling the behaviors of biological synapses. Continuous conductance switching can be achieved by adjusting the compliance current during the SET process and the stop voltage amplitude of RESET. A higher compliance current yields a more robust conductive filament via enhanced oxygen vacancy generation, thereby increasing the device conductance. Conversely, a higher RESET stop voltage truncates the filament more severely, resulting in the gradual RESET behavior observed in Figure 2a. In devices with active metal electrodes (such as Ag or Cu), the conductive filaments are predominantly formed from the electrode metal atoms themselves [144]. For instance, in Ag‐based memristive devices, the SET process is initiated by a positive bias that oxidizes the anode. The resulting Ag^+^ ions migrate to the cathode, where they are reduced to form a conductive metallic filament. Conversely, a reverse bias dissolves the filament, resetting the device to the HRS (Figure 2b), thereby completing the switching cycle [145].

#### 2.1.2 Carrier Trapping/Detrapping

The resistive switching in purely electronic memristors primarily results from the trapping and release of carriers at defects within the material. These defects can be intentionally introduced through external doping, such as nanoparticles [146, 147] or quantum dots [148, 149, 150]. Furthermore, they may arise from intrinsic material defects, including oxygen vacancies [151] or sulfur vacancies [152]. The resistive switching in purely electronic memristors does not involve ion migration. This mechanism significantly reduces the structural damage caused by electrical signals to the device microstructure. Consequently, compared to conductive filament‐type memristors, purely electronic memristors feature superior device uniformity and operational stability. Their operating mechanisms primarily include space charge limited current (SCLC) [153, 154, 155] and Poole‐Frenkel (P‐F) [156, 157, 158]. These mechanisms directly influence carrier transport within the device, acting either independently or synergistically to achieve resistance regulation. Each mechanism will be discussed in the following sections.

SCLC is a prevalent bulk transport mechanism in electronic memristors [159]. When a large density of charge traps exists within the material, the injection and capture of charge carriers are modulated by space‐charge effects. At low electric fields, injected electrons are captured by deep traps. The resulting scarcity of free carriers leads to a low current, corresponding to the HRS. As the electric field increases, these traps become progressively filled. The consequent rise in free‐carrier concentration drives a sharp increase in current, switching the device to the LRS. A reverse voltage releases the trapped electrons, resetting the device to the HRS.

This mechanism can be exemplified by ZnO‐based devices, where oxygen vacancies and zinc interstitials act as key intrinsic donor defects [160]. The energy level of oxygen vacancies lies below that of zinc interstitials in ZnO. Therefore, in the SET process, injected electrons are preferentially captured by oxygen vacancies and subsequently by zinc interstitials. During RESET, electrons trapped at oxygen vacancies and zinc interstitials are released under an applied reverse electric field (Figure 2c). In addition, the electrons trapping and detrapping process can be controllably adjusted by regulating the current compliance values or voltage amplitudes.

P‐F emission is another volume conduction mechanism commonly observed in medium materials with high trap density and sufficient thickness to suppress tunneling. An external electric field can reduce the height or width of the potential barrier associated with traps under the P‐F mechanism, facilitating the transition of electrons to the conduction band upon thermal excitation. For example, the high trap density formed by oxygen vacancies promotes P‐F emission to dominate in high‐k HfO_2_ [161, 162, 163]. Yoon et al. reported a Pt/Ta_2_O_5_/HfO_2_/TiN device where electrons are initially captured by deep traps, resulting in a HRS as voltage increases [164]. Subsequently, shallow traps dominate transport, and the effective trap depth decreases, driving the device into the LRS (Figure 2d).

#### 2.1.3 Phase‐Change Memristors

Phase‐change memristors harness the reversible transition between amorphous and crystalline phases—two states that exhibit distinct electrical resistivities [165]. The mechanism relies on Joule heating induced by electrical pulses. When a longer, lower‐amplitude pulse is applied, the phase‐change material is heated above the recrystallization temperature but below the melting point; upon slow cooling, it forms a low‐resistance crystalline state (SET). Conversely, when a short, high‐amplitude pulse is applied, the temperature rises above the melting point; upon rapid quenching, the phase‐change material amorphizes, resetting the device to a high‐resistance state (RESET). The pronounced contrast in resistivity between the two phases—often exceeding three orders of magnitude—enables reliable non‐volatile data storage and analog switching behavior through partial crystallization [103, 107, 166, 167, 168].

Ge_2_Sb_2_Te_2_ (GST) [107, 165, 168, 169] and GeTe‐based alloys [103, 166, 167] remain the most extensively studied material systems, owing to their favorable crystallization kinetics and thermal stability. Structurally, phase‐change memristor cells typically adopt a mushroom‐type configuration, in which a bottom electrode constricts the current to a small volume of phase‐change material for efficient thermal confinement. For instance, Song et al. proposed a GST phase‐change memristor with a mushroom‐type nano‐confined cell structure that enables RESET switching with reduced program energy [169]. This reduction in programming energy mitigates the over‐programming effect caused by excessive RESET current, extending the endurance of the phase‐change memristors beyond 1.1×10^11^ switching cycles.

#### 2.1.4 Ferroelectric Memristors

Ferroelectric memristors leverage the polarization reversal in ferroelectric materials to modulate the device conductance [170]. The key mechanism involves the polarization‐dependent modulation of the interface potential barrier or the distribution of charge carriers. When an electric field exceeding the coercive voltage is applied, the spontaneous polarization in the ferroelectric layer switches direction, altering the band alignment at the metal‐ferroelectric interface or affecting the depletion width in a semiconductor layer. This results in a non‐volatile change in resistance without requiring long‐distance transport of atoms, enabling ultrafast switching (sub‐nanosecond) and exceptionally low energy consumption [108, 171, 172, 173]. The device conductance is governed by the history of the applied polarization states, allowing continuous, analog resistance tuning through partial polarization switching [108]. This reversible, non‐destructive readout capability, combined with analog switching behavior, enables applications in neuromorphic computing and high‐density memory arrays [173].

Typical device architectures include metal‐ferroelectric‐metal capacitors where the ferroelectric layer itself serves as the switching medium, and metal‐ferroelectric‐insulator‐semiconductor structures that incorporate a dielectric barrier to enhance the resistance contrast. Perovskite oxides such as BiFeO_3_ [170], Pb(Zr,Ti)O_3_ [171], and BaTiO_3_ [108, 173], as well as doped HfO_2_ [172], have been widely adopted. For instance, Ma et al. reported a high‐performance memristor based on a BaTiO_3_/Nb:SrTiO_3_ ferroelectric tunnel junction, featuring fast operation speed (600 ps) and high state density (5 bits) [173]. The device exhibits sub‐nanosecond resistive switching with a low write current density. Additionally, ultrafast spike‐timing‐dependent plasticity—a key synaptic function—was demonstrated. Beyond oxide ferroelectric materials, ferroelectric polymers have also attracted considerable research interest owing to their intrinsic analog switching behavior and excellent flexibility [174].

#### 2.1.5 Magnetic Memristors

Magnetic memristors utilize spin‐dependent transport to achieve non‐volatile resistance switching [175, 176, 177]. The operating principle is primarily based on the magnetic tunnel junction structure, which consists of two ferromagnetic layers separated by a thin insulating barrier [178]. One ferromagnetic layer has a fixed magnetization direction (the reference layer), while the other is free (the free layer). The relative alignment of the magnetizations—parallel or antiparallel—dictates the junction resistance due to the tunnel magnetoresistance effect. The memristive behavior arises from the ability to modulate the free‐layer magnetization with an electric current or voltage. In spin‐transfer torque devices, a spin‐polarized current exerts torque on the free‐layer magnetization, enabling deterministic switching.

More recent implementations employ spin—orbit torque, in which an in‐plane current generates a spin current via the spin Hall effect, enabling faster switching and improved endurance [179]. The resistance can be tuned to multiple intermediate states by varying the current amplitude or pulse duration, which controls magnetization dynamics and enables analog, history‐dependent electrical behaviors. Magnetic memristors offer excellent endurance, high speed, and compatibility with traditional CMOS processing, positioning them as main candidates for non‐volatile memory and synaptic devices in neuromorphic circuits. For instance, Zhang et al. demonstrated a nanoscale spin‐torque memristor based on a perpendicular‐anisotropy magnetic tunnel junction with a CoFeB/W/CoFeB composite free layer [177]. It exhibits a tunneling magnetoresistance exceeding 200%. Memristive behavior is achieved through spin‐transfer torque switching with the resistance states retained by strong domain wall pinning effects in the free layer. Furthermore, some important synaptic functions such as spike‐timing‐dependent plasticity are simulated. Beyond simulating synaptic functions, magnetic memristors can also be used to construct artificial neurons. For example, Torrejon et al. demonstrated an oscillatory neuron using a FeB/MgO/CoFeB magnetic tunnel junction [180]. Leveraging its nonlinear oscillation dynamics to mimic neuronal pulse behaviors, this device can be used to achieve spoken‐digit recognition.

### 2.2 Optoelectronic Memristors

Optical signals introduce several advantages for advancing memristor technology. Conventional memristors, which are electrically controlled by voltage or current signals, rely on ion migration to modulate conductance—a process that inevitably alters the device microstructure. Furthermore, the relatively high operating voltages or currents required often elicit substantial Joule heating, which aggravates microstructural changes and ultimately degrades operational stability. In recent years, extensive research efforts have focused on improving memristor stability, resulting in significant enhancements through optimization of materials and device structures. In contrast to electrical inputs, optical signals offer ultrahigh speed and broad bandwidth. At low optical power densities, they can modulate conductance without substantially perturbing the microstructure or generating Joule heat. Thus, optical tuning of memristor conductance may be considered a nearly non‑destructive approach, offering a promising route to overcome stability limitations. Moreover, optoelectronic memristors can directly detect, process, and store optical information in situ within the same device. This intrinsic functionality makes them attractive for emerging applications such as artificial vision systems that integrate sensing, memory, and computing, as well as for optoelectronic neuromorphic computing architectures [181, 182, 183]. Consequently, optoelectronic memristora have attracted increasingly widespread attention from researchers [184, 185, 186, 187]. Currently, optoelectronic memristors can be categorized into two main types based on their operation mode: light‐electricity synergistic control and all‐optical control (AOC).

#### 2.2.1 Light‐Electricity Synergistic Control

The reversible modulation of memristor conductance is essential for realizing efficient brain‑inspired computing. Light‑electricity synergistic control refers to a class of devices whose conductance tuning relies on the combined action of both optical and electrical signals. Depending on the specific manner of control, such devices can be further classified into four categories: optical potentiation with electrical depression, electrical potentiation with optical depression, light‑assisted electrical control, and electricity‑assisted optical control.

##### 2.2.1.1 Optical Potentiation With Electrical Depression

**FIGURE 3:**
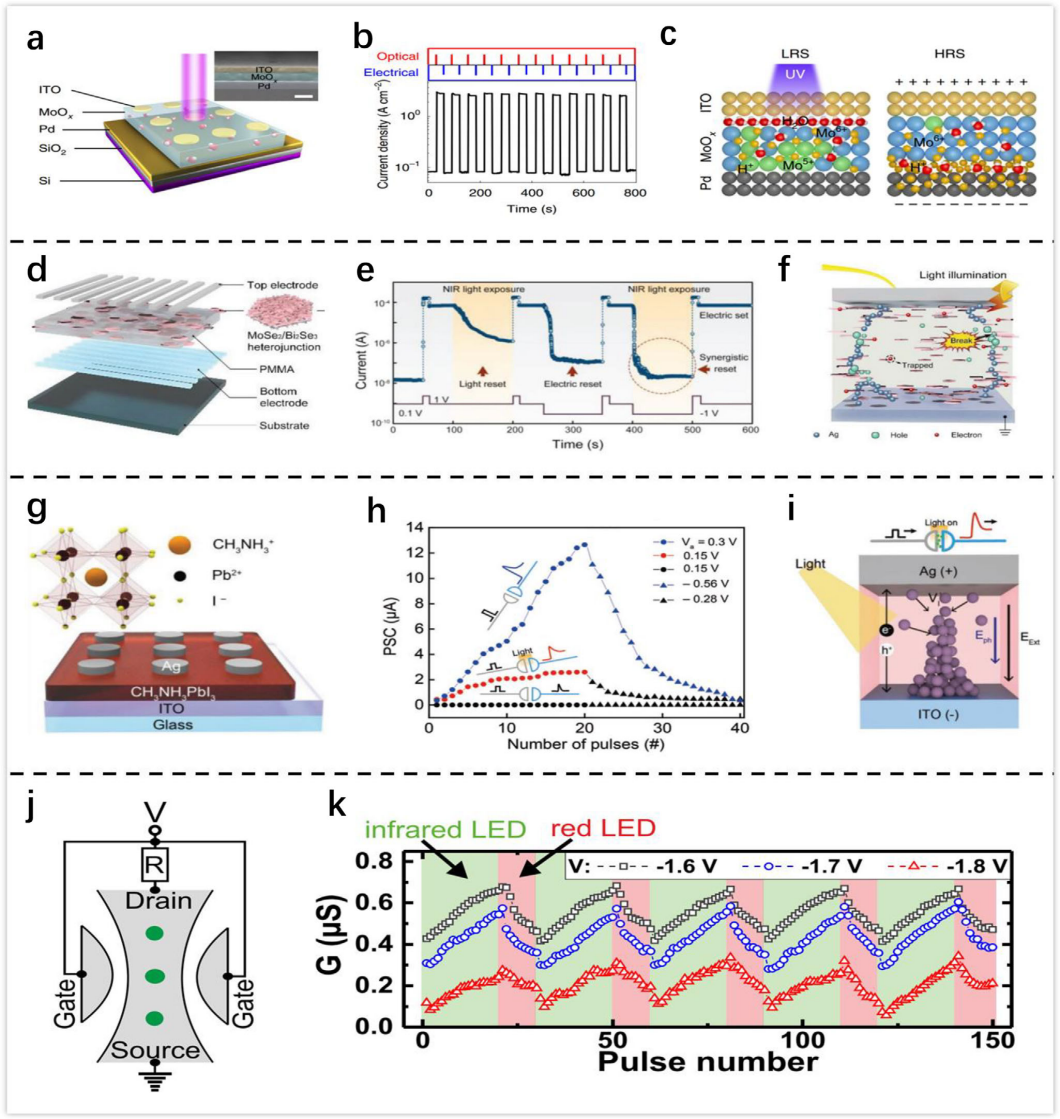
(a–c) Optical potentiation and electrical depression. (a) Schematic illustration of the ITO/MoO_x_/Pd device. (b) Optical potentiation and electrical depression. (c) Schematic illustration of the resistive switching mechanism for the ITO/MoO_x_/Pd device. Reproduced with permission [188]. Copyright 2019, Springer Nature. (d–f) Electrical potentiation and optical depression. (d) Schematic illustration of the MoSe_2_/Bi_2_Se_3_ nanosheets heterostructure. (e) Electrical potentiation and optical depression. (f) Schematic illustration of the resistive switching mechanism for the MoSe_2_/Bi_2_Se_3_ nanosheets heterostructure. Reproduced with permission [190]. Copyright 2019, Wiley‐VCH. (g–i) Light‐assisted electrical control. (g) Schematic illustration of the MAPbI_3_‐based device. (h) Light‐Assisted electrical control. (i) Schematic illustration of the resistive switching mechanism for the MAPbI_3_‐based memristor. Reproduced with permission [192]. Copyright 2018, Wiley‐VCH. (j, k) Electricity‐Assisted Optical Control. (j) Schematic illustration of an InAs quantum dot‐based memristor. (k) Electricity‐Assisted optical control. Reproduced with permission [193]. Copyright 2016, AIP Publishing.

In optoelectronic memristors that exhibit optical potentiation and electrical depression, conductance is enhanced by light and reduced by electrical signals. This behavior is often based on the persistent photoconductivity (PPC) effect, whereby exposure to light increases the conductance of the device—an elevated state that persists long after illumination ceases, typically owing to defect states in the materials. For instance, Zhou et al. demonstrated a memristor capable of optical potentiation and electrical depression with a Pd/MoO_x_/ITO structure (Figure 3a) [188]. It can be returned to HRS by applying a negative voltage for the next operation cycle (Figure 3b). Stable cycling is achieved using a light pulse (600 ms) for SET and an electrical pulse (−4.5 V, 100 ms) for RESET. The switching mechanism involves a light‐induced valence change: UV illumination reduces Mo^6+^ to Mo^5+^ in MoO_x_. The resulting holes decompose adsorbed water molecules to release H^+^, which subsequently forms conductive H_y_MoO_3_. During the electrical RESET, the applied electric field drives H^+^ ions out of the active layer, restoring the original valence state and returning the device to HRS (Figure 3c).

##### 2.2.1.2 Electrical Potentiation With Optical Depression

Unlike the above‐mentioned UV light potentiation mechanism, long‐wavelength light exhibits the potential to decrease device conductance in some conductive filament‐type memristors. In such systems, a conductive filament is typically first formed under an electrical signal to bring the device into LRS; subsequent optical exposure then reduces the conductance of the memristors [189, 190, 191]. Wang and colleagues developed a near‐infrared (NIR) light‐controlled memory device based on a MoSe_2_/Bi_2_Se_3_ heterostructure with a Ag electrode (Figure 3d), which employs a control mode of electrical potentiation and optical depression to mimic synaptic plasticity (Figure 3e) [190]. In the dark, application of a SET voltage induces the formation of a Ag conductive filament, switching the device from HRS to LRS. When exposed to 790 nm NIR light, the generated photogenerated holes break the Ag filament, corresponding to the optical RESET process (Figure 3f).

##### 2.2.1.3 Light‑Assisted Electrical Control

When the electric field induced by light aligns with the device's external electric field, the energy barrier for the migration of charge carriers or ions is effectively lowered. This alignment not only reduces the electrical threshold required for device switching but also enhances the overall control efficiency. The study by Wang et al. demonstrated a memristor device based on MAPbI_3_ that enables light‐assisted electrical control (Figure 3g) [192]. This effect arises from applying a positive bias to the top electrode, which aligns the photo‐induced field (E_ph_) and the external field (E_xt_). Driven by this cooperative effect, the migration of iodine vacancies is markedly enhanced (Figure 3i). A voltage pulse of 0.15 V is sufficient to increase the device's conductance under illumination, while a voltage pulse of −0.28 V is required to decrease the conduction under dark conditions. This indicates that introducing light exposure can lower the resistive switching voltage of the device, achieving reduced operating power consumption.

##### 2.2.1.4 Electricity‑Assisted Optical Control

Beyond modulating electrical behavior with light, achieving precise optical control through electrical means represents an important advance toward sophisticated optoelectronic neuromorphic systems with higher dimensionality and tunability. Along this direction, Hartmann and co‐workers developed an electricity‐assisted optical control memristor (Figure 3j), which is based on a GaAs/AlGaAs heterojunction where InAs quantum dots (QDs) grow within fixed‐position holes [193]. This device features four ports with additional gates on both sides. The gates are connected to the drain of the device for voltage application. The introduction of InAs QDs changes the band structure at the heterojunction interface. GaAs/AlGaAs heterojunctions generate electron‐hole pairs under light exposure, allowing QDs to charge or discharge without an applied bias. The degree of optical response in the device is influenced by the magnitude of the applied bias (Figure 3k).

#### 2.2.2 All‐Optical Control

The conductance of an AOC memristor can be reversibly modulated through optical stimulation, thereby avoiding microstructural changes caused by ion migration and Joule heating from electric fields or currents. Neuromorphic devices utilizing AOC memristors are viewed as promising candidates for future visual sensors that integrate sensing, computing, and memory capabilities [94, 194, 195, 196, 197, 198, 199, 200].

**FIGURE 4:**
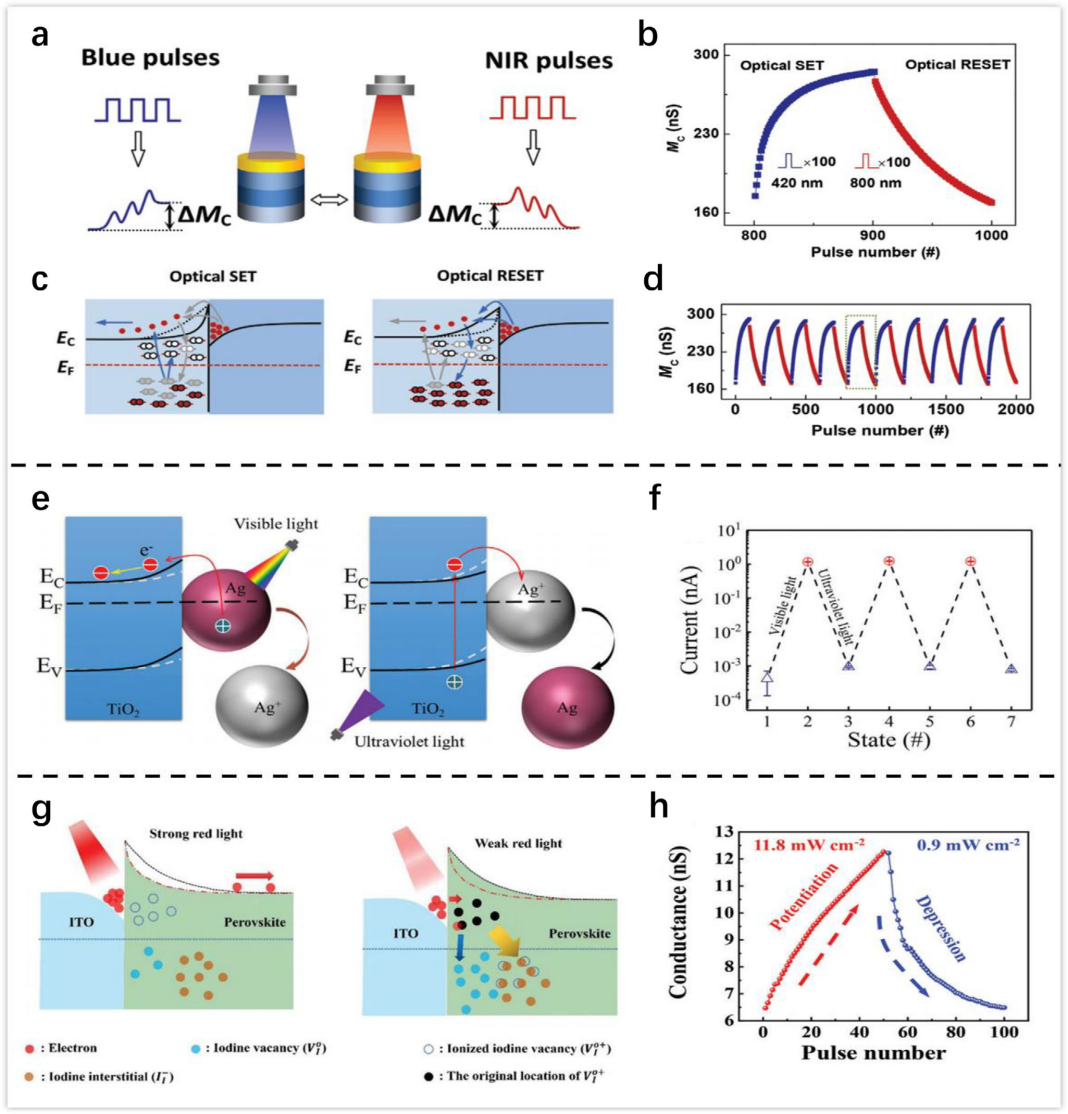
AOC memristors. (a) Schematic illustration of the IGZO‐based AOC memristor. (b) Modulating device conductivity with optical wavelength. (c) Schematic illustration of the resistive switching mechanism for the IGZO device. (d) Reversible conductance regulation under blue and NIR light illumination. Reproduced with permission [195]. Copyright 2020, The Authors, published by Wiley‐VCH under the terms of the Creative Commons Attribution (CC BY) License (a–d). (e) Schematic illustration of the resistive switching mechanism for the Ag‐TiO_2_ nanocomposite film. (f) Reversible conductance regulation under visible and ultraviolet light. Reproduced with permission [210]. Copyright 2020, Wiley‐VCH (e, f). (g) Schematic illustration of the resistive switching mechanism for the Au/Cs_x_FA_y_MA_1‐x‐y_Pb(IzBr_1‐z_)_3_/ITO/PEN device. (h) Modulating device conductivity with optical intensity. Reproduced with permission [206]. Copyright 2023, Wiley‐VCH (g, h).

However, the implementation of AOC memristors faces challenges due to the inherent photoelectric effect of semiconductors [201]. In 2020, Zhuge's team developed the first AOC memristor based on InGaZnO (IGZO) and utilized it to simulate synaptic functions (Figure 4a) [195, 202]. This memristor enables reversible conductance modulation simply by adjusting the wavelength of the optical signal (Figure 4b). The reversible conductance tuning is attributed to light‐induced dynamic changes in the width of the interfacial barrier within the bilayer oxide. Specifically, the ionization of oxygen vacancies becomes the primary process under short‐wavelength light, increasing the number of ionized oxygen vacancies at the interface. As a result, the interfacial barrier narrows, enhancing the tunneling current and increasing the device's conductance (Figure 4c). In contrast, the neutralization of oxygen vacancies is more pronounced than ionization under long‐wavelength light. A decrease in ionized vacancy concentration leads to a widening of the interfacial barrier, which weakens the tunneling current. Figure 4d shows the reversible conductance modulation of the device: conductance increases under 420 nm light pulses and decreases under 800 nm light pulses.

Furthermore, researchers have demonstrated a neuromorphic vision sensor with in‐situ cryptographic computing capability based on the IGZO‐AOC memristor [182]. Notably, a single‑layer ZnO memristor fabricated by sputtering the oxide film in pure argon also exhibited AOC behaviors [194]. Using this AOC memristor, complete Boolean logic functions have been successfully implemented. In recent years, AOC memristors have attracted significant research interest. Building on these insights, a variety of AOC memristors have emerged, with implementations spanning metal oxides [94, 194, 198, 203], 2D materials [204], perovskites [205, 206, 207], and organic materials [197, 208, 209].

For example, Shan et al. developed an Ag‐TiO_2_ based plasmonic optoelectronic memristor [210]. This device features a Au/Ag‐TiO_2_/FTO sandwich structure, where Ag nanoparticles are uniformly distributed within the TiO_2_ matrix and exhibit pronounced localized surface plasmon resonance absorption characteristics. This device exhibits all‐optically controlled synaptic plasticity: under visible light stimulation, it demonstrates excitatory postsynaptic currents that induce long‐term potentiation; whereas under UV irradiation, it generates inhibitory postsynaptic currents that achieve long‐term depression. This is because visible light excitation generates hot electrons in Ag nanoparticles, which inject into the conduction band of TiO_2_ and promote the oxidation of Ag^0^ to Ag^+^, resulting in enhanced conductivity. Ultraviolet light, however, excites interband transitions in TiO_2_, facilitating the reduction of Ag^+^ back to Ag^0^ and causing a decrease in conductivity (Figure 4e). By applying visible and UV light pulses, the device achieves reversible modulation of synaptic weights, demonstrating stable optical control with good cycling stability (Figure 4f).

Compared to wavelength‐modulated conductivity, achieving reversible conductance regulation through variations in light intensity represents another form of AOC. The study by Cai et al. reported an AOC memristor based on the perovskite structure, which achieves reversible conductance regulation by adjusting the intensity of red light (630 nm) exposure [206]. The ionization effect of iodine vacancies dominates under strong light (11.8 mW cm^−2^), leading to increased concentrations of ionized iodine vacancies in the interface region. This narrows the interface potential barrier and enhances tunneling current, thereby increasing the device conductance. Conversely, the recombination effect between V^+^ and iodine ions (I^−^) surpasses ionization under weak light (0.9 mW cm^−2^). This reduces the concentration of V^+^ and widens the interface barrier, which decreases the device conductance (Figure 4g,h).

## 3 Electronic Memristive RC

Over the past decade, memristor‐based RC systems have undergone rapid development in both architecture and functionality. These systems exhibit superior performance in image recognition [125, 211, 212], speech recognition [36, 66, 213, 214, 215, 216], and sequence prediction [50, 65, 217, 218]. Electronic memristors offer the advantage of performing signal injection, processing, and readout entirely within the electrical domain. Their operational mechanisms are compatible with CMOS, enabling the fabrication of crossbar arrays and providing a hardware foundation for high‐dimensional reservoirs with numerous virtual nodes.

### 3.1 Pattern Recognition

#### 3.1.1 Image Recognition

**FIGURE 5:**
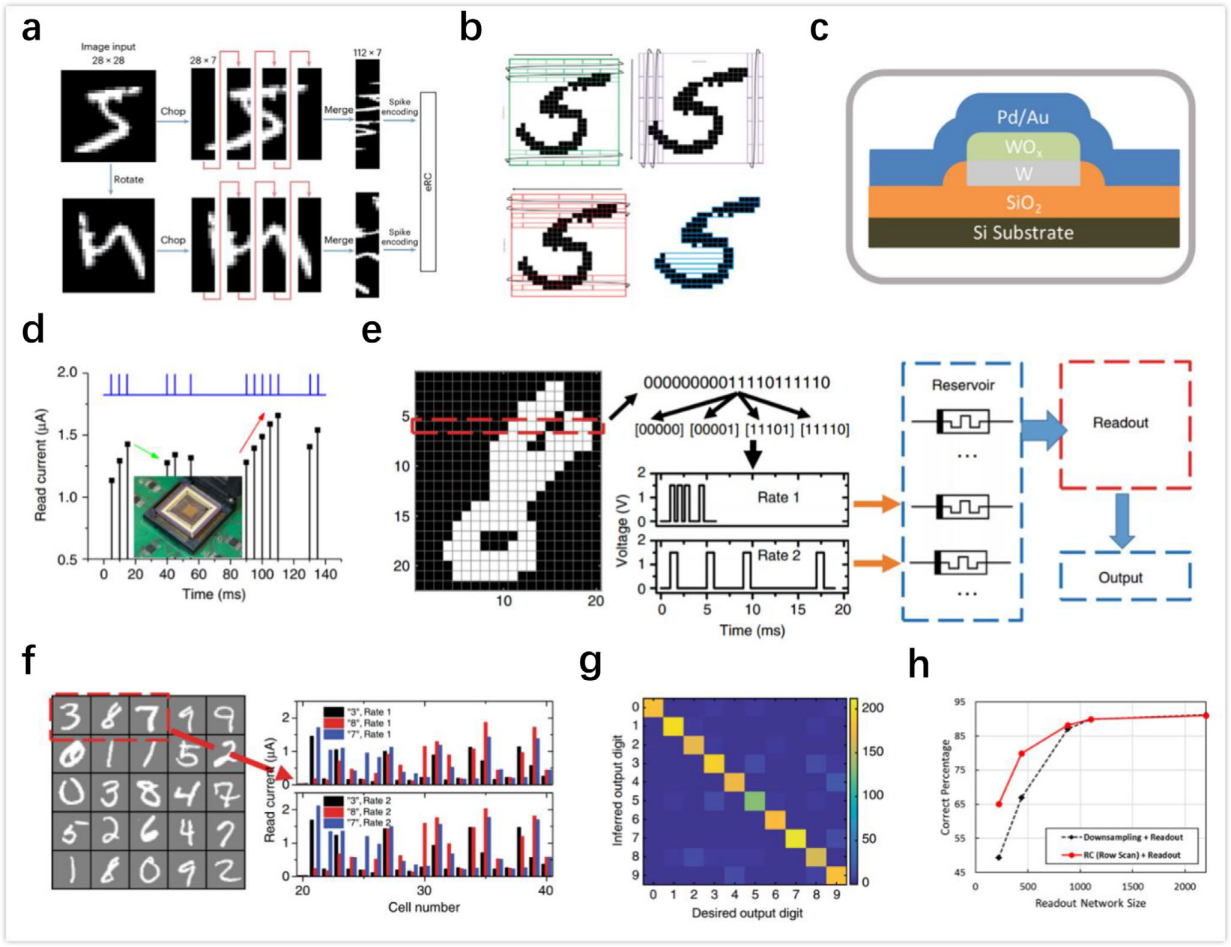
RC system for image recognition. (a) Common preprocessing methods for MNIST handwritten images. Reproduced with permission [26]. Copyright 2024, Springer Nature. (b) Multi‐directional image pixel transformation scheme: Green, mask length: 4, direction: left to right; Purple, mask length: 5, direction: up to down; Red, mask length: 5, direction: up to down; Blue, mask length: random, direction: up to down. Reproduced with permission [120]. Copyright 2025, Elsevier. (c–h) Handwritten digit recognition using a memristor‐based RC system. (c) Device structure and (d) response to a pulse stream with different time intervals. (e) Transformation of image pixels into input pulses with different frequencies. (f) Reservoir states corresponding to the three examples at two input rates and (g) recognition results. (h) Recognition performance between RC systems and traditional networks. Reproduced with permission [65]. Copyright 2017, The Authors, published by Springer Nature under a Creative Commons Attribution 4.0 International License.

Currently, image recognition tasks typically rely on complex convolutional operations for feature extraction, which demand substantial hardware resources and are unfavorable for rapid data processing. In contrast, memristor RC networks can efficiently extract image features by leveraging the intrinsic dynamic behaviors of the devices, thereby significantly reducing hardware overhead. Specifically, converting image pixels into electrical pulses and feeding them into a memristor enables the device to extract input information through the final or intermediate reservoir states. Subsequently, the reservoir states are fed into the readout layer for recognition. However, challenging image recognition tasks necessitate that memristors exhibit sufficiently separable reservoir states to ensure that recognition accuracy meets requirements. Employing appropriate coding methods to enhance reservoir state diversity is usually effective under conditions of limited reservoir space. For example, chopping, merging, and rotating the images prior to spike coding can increase the signal at the input channel while shortening the length of each sequence in the case of handwritten digit images, thereby preventing reservoir capacity overload (Figure 5a) [26, 85, 219].

In addition, Jin et al. introduced a distinctive feature extraction method that combines multi‐length masks and multi‐directional sampling strategies to further enhance features from images (Figure 5b) [120]. In the green graph, the single‐input mask length is set to 4, performing image sampling from left to right; in the purple graph, the single‐input mask length is set to 5 for top‐to‐bottom sampling; and in the red graph, it is set to 7 for right‐to‐left sampling. In the case of the blue graph, the sampling area for each image row corresponds to the distance between the two farthest black pixels in that row, and the input mask length is the minimum span of all black points in the respective row. This cross‐row strategy enables a focus on a distinct range with inputs of different lengths, thus producing different reservoir states. Moreover, the different sampling directions captured varied information. This sampling strategy effectively extracts diverse features, providing more comprehensive information for MNIST.

Optimizing the key parameters of encoded electrical pulses, such as frequency [65, 220, 221], amplitude [36, 56, 79, 215, 222], and pulse width [121, 223, 224], can also enrich the reservoir states and directly improves state separation in RC systems. In 2017, Lu and colleagues demonstrated an RC system based on the Pd/Au/WO_x_/SiO_2_/W memristor (Figure 5c) [65]. Specifically, when an electrical pulse is applied, the device's conductance state increases due to the formation of nanoscale conductive filaments containing oxygen vacancies or metallic particles. Upon removal of the electric bias, these oxygen vacancies or metal particles gradually redistribute to their initial steady state due to spontaneous recombination/diffusion, resulting in nonlinear dynamic conductivity decay. Therefore, applying several consecutive pulses with short intervals causes the conductance state to progressively increase, whereas a long interval between pulses allows conductance to decay back to its initial state (Figure 5d).

For MNIST handwritten digit recognition, two complementary strategies were employed to increase accuracy (Figure 5e). One strategy is to reduce the single‐input sequence length to decrease the recognition capacity required for each memristor. Although this can improve the recognition accuracy of the RC system, the number of virtual nodes will increase, leading to decreased read efficiency of the system. Another strategy involves adjusting the frequency of the input pulse, as the interval between pulses affects the final conductance state due to the relaxation process. Figure 5f shows the reservoir states measured for the three samples at two distinct input rates, illustrating that the reservoir enables effective differentiation of the inputs. This demonstrates how the intrinsic relaxation dynamics of the device—governed by its characteristic time constant—translate into a computational advantage. Varying the pulse interval effectively samples the memristor's fading memory across different time scales. Inputs that drive the device into distinct regimes along its nonlinear relaxation trajectory yield well‐separated final conductance states.

The reservoir states are then fed into the readout function for classification. Figure 5g presents the confusion matrix, highlighting the comparison between the classification results obtained from the RC system experiments and the expected outputs. The authors contrasted the MNIST recognition accuracy of the RC system with that of traditional networks at identical network sizes, demonstrating the RC system's efficient computational capabilities (Figure 5h).

**FIGURE 6:**
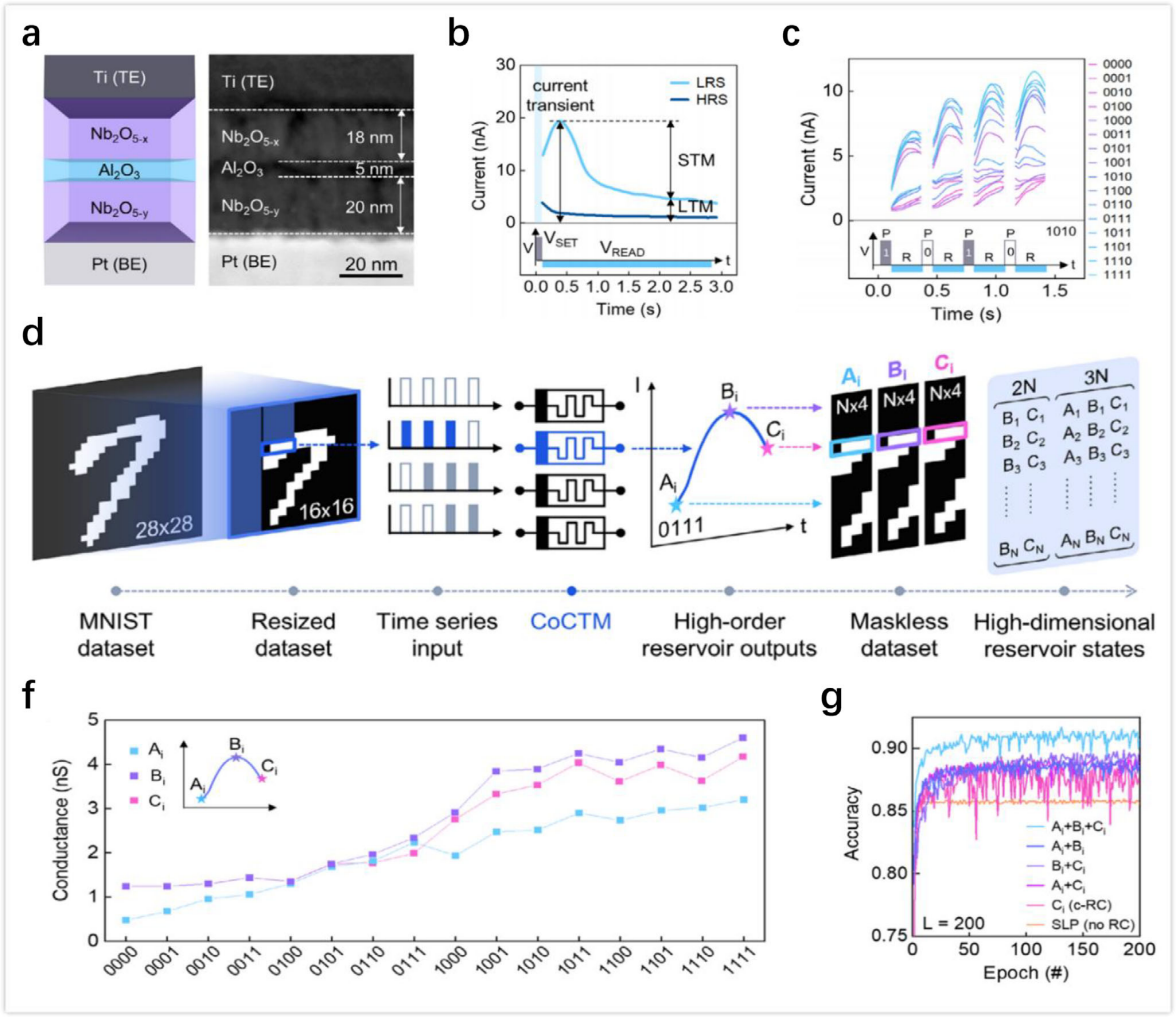
Recognition of MNIST handwritten images by high‐dimensional RC systems based on electrical memristors. (a) Schematic illustration of the Ti/Nb_2_O_5−x_/Al_2_O_3_/Nb_2_O_5−y_/Pt device. (b) Current transient during a V_READ_ pulse following a writing pulse. (c) Current transient responses to 16 combinations of 4‐bit pulse sequences. (d) Schematic diagram of high‐dimensional RC system. (e) Reservoir states extracted from the initial (Ai), peak (Bi), and final (Ci) currents in the transient. (f) Recognition accuracy of the RC system over 200 epochs of training for various combinations of reservoir states. Reproduced with permission [82]. Copyright 2025, The Authors, published by Wiley‐VCH under the terms of the Creative Commons Attribution (CC BY) License.

In addition to pulse parameter regulation, extending reservoir state nodes using higher‐order time responses is also a common method for enhancing RC recognition capabilities. For instance, Martinez et al. reported a memristor‐based RC device with a Ti/Nb_2_O_5‐x_/Al_2_O_3_/Nb_2_O_5‐y_/Pt structure (Figure 6a) [82]. After a programming pulse (10 V, 100 ms), a subsequent read voltage (−2.5 V) elicits a rapid current increase followed by double‐exponential decay, indicative of short‐term memory (STM) dynamics (Figure 6b). This intrinsic transient behavior allows the device to encode multi‐bit input sequences into high‐dimensional state space: 16 distinct 4‐bit pulse combinations each produce separable current trajectories, highlighting its suitability for temporal signal processing (Figure 6c).

By integrating these higher‐order memristive dynamics into an RC framework (Figure 6d), the authors constructed a rich state space using three features extracted from the current response: the initial current (A_i_), peak current (B_i_), and steady‐state current (C_i_) (Figure 6e). In MNIST handwritten digit classification, this high‐dimensional RC model—simultaneously leveraging A_i_, B_i_, and C_i—_achieved an accuracy of 0.917, surpassing the 0.895 accuracy of a conventional RC model under the same network scale (Figure 6f). The improvement arises from the enhanced separability of reservoir states enabled by the device's higher‐order dynamics, which preserves classification performance even as the input image dimensionality is reduced.

**FIGURE 7:**
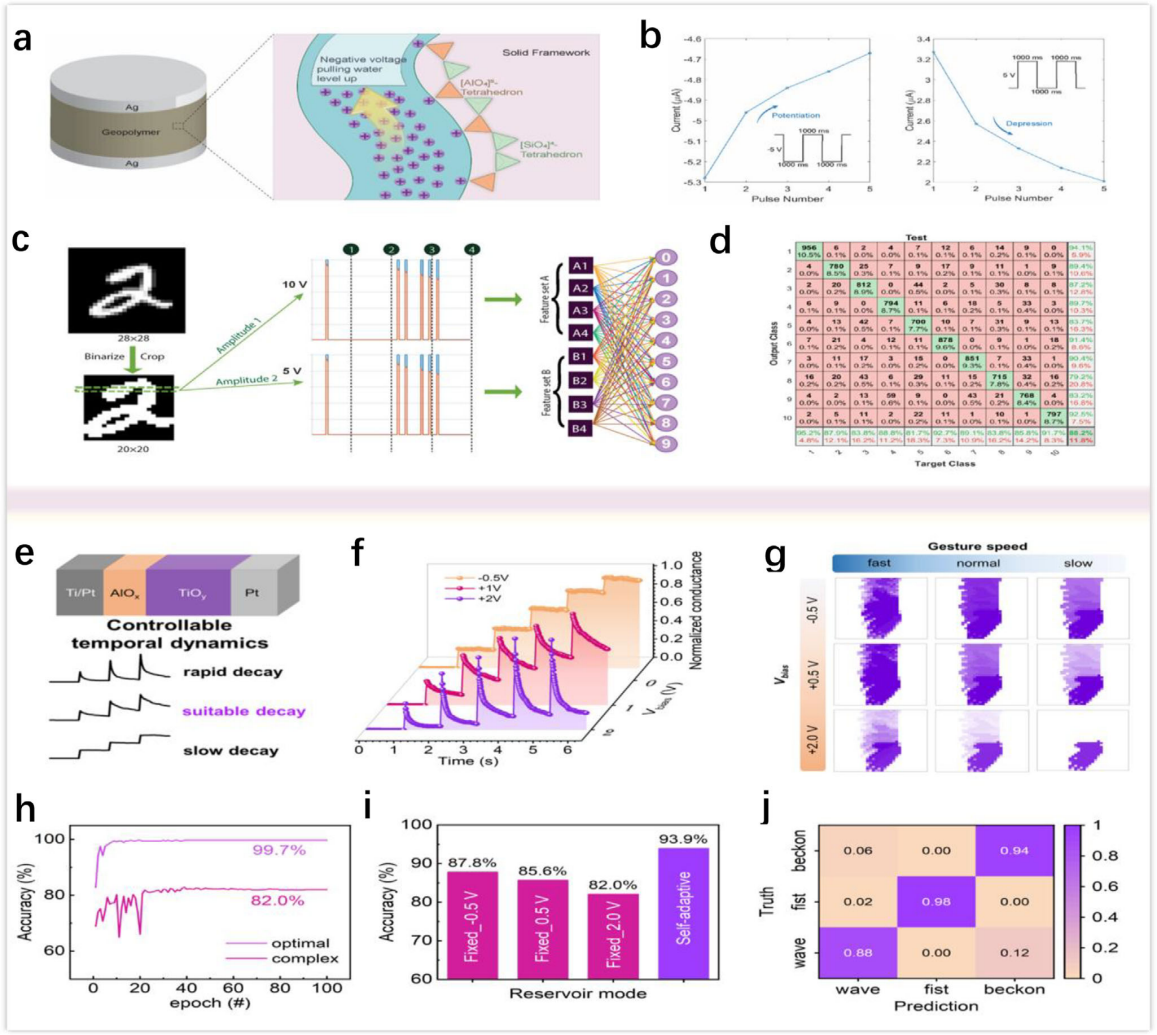
(a–d) Memristive RC system controlled using voltage amplitude. (a) Ag/Geopolymer/Ag memristor structure and magnified view of tortuous capillary. (b) The memristor achieves synaptic potentiation and depression through voltage amplitude modulation. (c) Transformation of image pixels into input pulses with different voltages. (d) Confusion matrix of MNIST handwritten digits. Reproduced with permission [222]. Copyright 2025, The Authors, published by AIP Publishing under a Creative Commons Attribution (CC BY) license. (e–j) A precise time‐constant modulation scheme based on controlling the static bias (V_bias_) for self‐adaptive RC system. (e) Schematic illustration of the Pt/AlO_x_/TiO_y_/Pt device. (f) Memory decay behavior and (g) the final reservoir state in the device under different V_bias_ conditions and gesture speed conditions. (h) Recognition accuracy for optimal and complex temporal conditions based on the V_bias_ fixed RC system. (i) Comparison of recognition accuracy for the complex time scale under different RC systems and (j) confusion matrix of the experimental recognition outcome under the self‐adaptive RC system. Reproduced with permission [79]. Copyright 2025, American Chemical Society.

Voltage modulation can effectively alter the dynamic response characteristics of electrical memristors. This enables the construction of reservoir spaces with varying temporal scales, enhancing the recognition accuracy of RC systems. Therefore, the same memristor can be used to construct RC system with different time scales by input pulses of varying amplitudes. For instance, Shakib et al. proposed a polymer memristor‐based physical RC system (Figure 7a), whose mechanism stems from the unique electro‐osmosis and ion migration behaviors within the material [222]. When a negative voltage pulse is applied, Na^+^ ions in the pore solution move upward driven by the electric field, pulling the solution column upward. This increases the effective cross‐sectional area of the conductive channel within the capillary, leading to an increase in device conductance. Conversely, the conductance decreases (Figure 7b).

They cropped and binarized 28 × 28 pixel MNIST handwritten digits into 20 × 20 arrays, converting each row into two pulse streams with distinct voltage amplitudes fed into memristors. Subsequently, they extracted four features from each output current and eighty features from each voltage amplitude at time steps (Figure 7c). Ultimately, this process enabled the readout network to utilize these features for recognition, producing a test accuracy of 88.2% (Figure 7d).

RC systems typically employ fixed reservoir nodes with finite‐time dynamics, limiting their ability to process sequences with complex temporal characteristics. Exploring the dynamically tunable characteristics of memristors represents a key direction for expanding RC applications [225, 226, 227, 228, 229, 230]. To address tasks with varying temporal characteristics, an adaptive RC system for dynamic gesture recognition based on TiO_x_/AlO_y_ is proposed (Figure 7e) [79]. The device exhibits unique dynamic attenuation properties under different bias control conditions (Figure 7f). The effective time constant of the TiO_x_/AlO_y_ memristor can be tuned by the applied bias, enabling the reservoir's temporal response window to dynamically align with the speed of the input gesture.

Facilitated by an optimal bias configuration, high‐precision gesture recognition is realized (Figure 7g). When confronted with complex real‐world dynamics, the system's recognition rate drops to only 82% under fixed bias conditions (Figure 7h). The introduction of an adaptive feedback module delivers a significant performance leap. The system dynamically adjusts the time constant of the reservoir nodes to match the speed of the input gesture, thereby significantly improving recognition accuracy from 82.0% to 93.9% under the same complex conditions (Figure 7i). This confirms the computational advantage of aligning the device's dynamic properties with the task's temporal structure. A mismatched time constant hinders the state separation, while an optimized one maximizes the richness and relevance of the reservoir states produced for the given input dynamics. The reliability of this improvement is further confirmed in the confusion matrix (Figure 7j).

**FIGURE 8:**
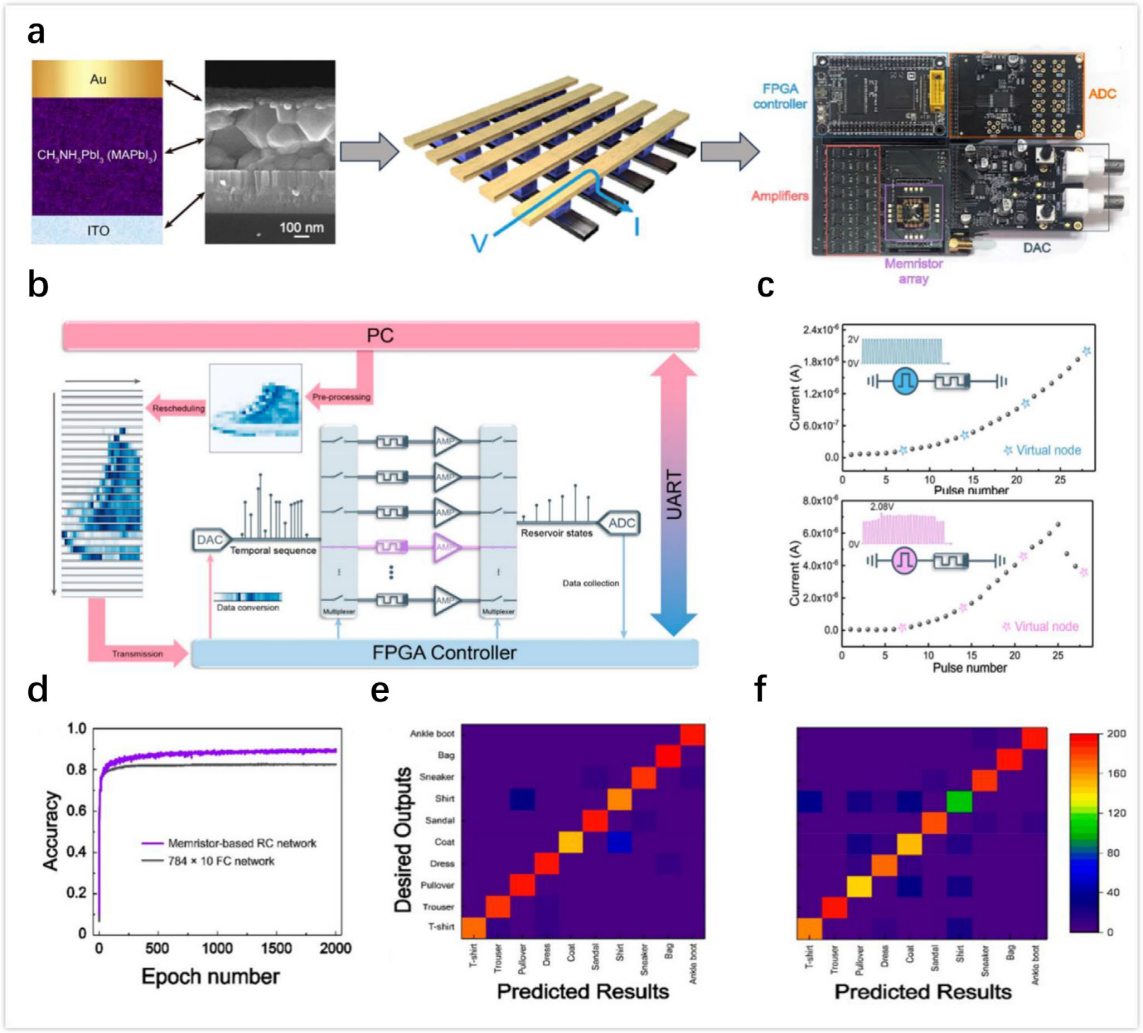
Memristive RC system based on voltage amplitude input for gray‐scale image recognition. (a) Schematic diagram of the structure and hardware integration of a MAPBI_3_‐based memristor. (b) Operational flowchart of the RC system for gray‐scale image recognition. (c) The reservoir state in the device under fixed voltage pulses and arbitrary voltage pulses. (d) Recognition accuracy on the Fashion‐MNIST dataset after 2000 epochs of training using the hardware RC system. (e) Confusion matrix showing the experimental classification results of the memristor‐based RC system and (f) software‐based neural network. Reproduced with permission [48]. Copyright 2022, American Chemical Society.

Currently reported memristor‐based RC networks predominantly employ binary encoding for images to reduce signal‐processing complexity. However, this approach inevitably introduces some degree of data distortion, thereby leading to a degradation in computational performance. To address this challenge, Yang and co‐workers demonstrated a hardware RC system based on MAPbI_3_‐based memristors for grayscale image recognition (Figure 8a) [48]. The system is broadly divided into five components: FPGA controller, amplifiers, memristor array, DAC module, and ADC module. The computer primarily handles data preprocessing and readout network training. The FPGA is used for circuit control and reservoir state acquisition. The memristor array serves as the physical reservoir. Figure 8b illustrates the operational flow of the memristor‐based RC system for classifying the Fashion‐MNIST dataset. The system generates an analog voltage pulse sequence from the grayscale values of a row of pixels in an image, inputs it into the memristor to achieve complex dynamic responses (Figure 8c), and ultimately feeds the output to the readout network for recognition. After 2000 training iterations, the hardware system achieved an impressive recognition accuracy of 90.1% (Figure 8d). Its confusion matrix clearly demonstrates precise differentiation among ten object classes (Figure 8e). In contrast, a purely software‐based fully connected neural network attained only 82.9% accuracy (Figure 8f), demonstrating the advantages of this memristor‐based physical reservoir in feature extraction and reducing training costs.

#### 3.1.2 Speech Recognition

In RC systems, encoding methods extend beyond image sampling and are especially effective for processing sequential signals like speech. Adjustable electrical pulse parameters—including amplitude, frequency, and duration—are mapped to distinct acoustic features, transforming raw audio inputs into a rich, time‐multiplexed pulse train. This encoding scheme allows the reservoir to nonlinearly project temporal inputs and extract discriminative patterns for various recognition tasks.

**FIGURE 9:**
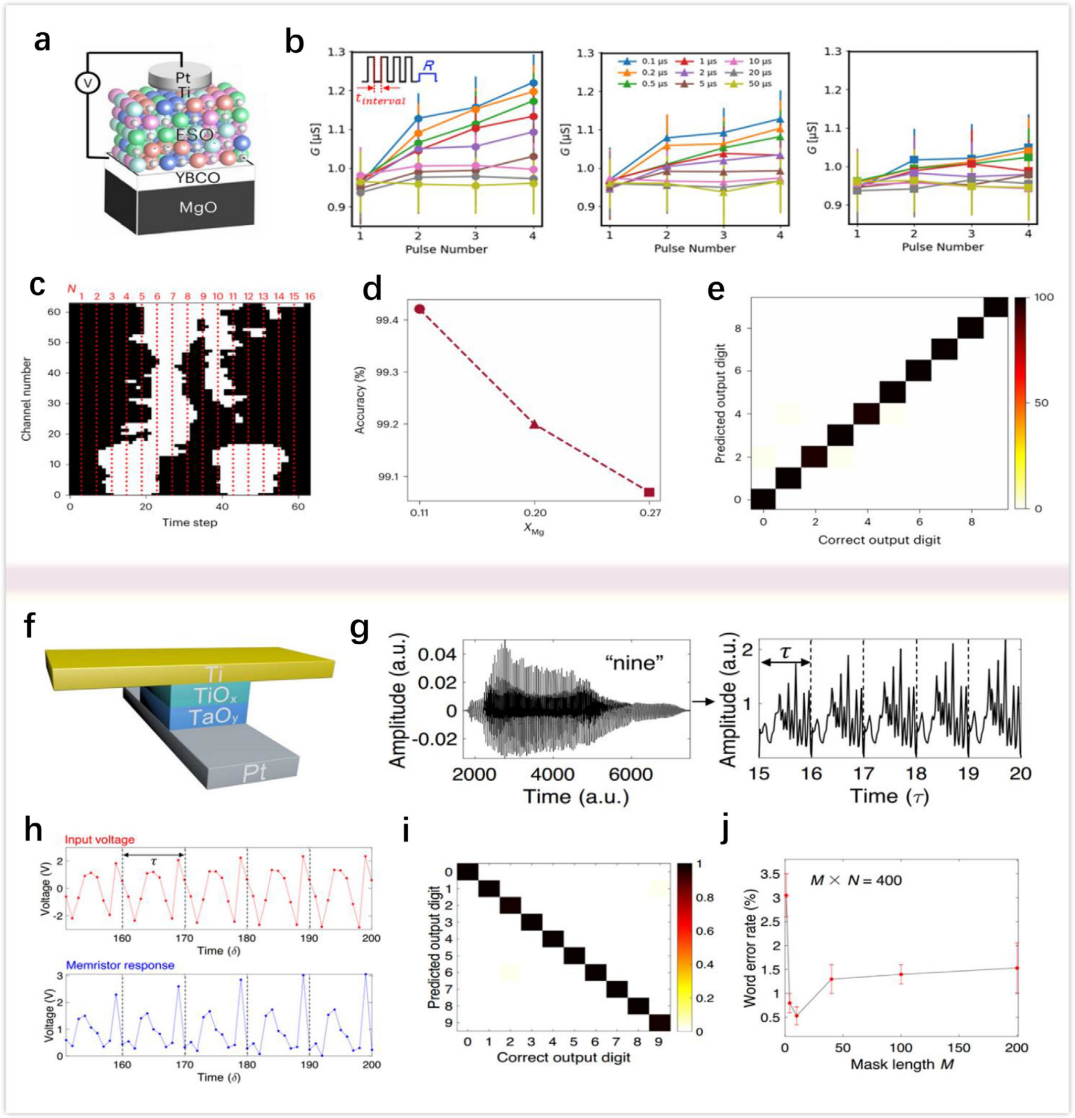
(a–d) Tunable ESO memristor‐based RC system for spoken‐digit recognition. (a) Schematic structure of the ESO memristor. (b) Conductance changes induced by pulse trains on ESO‐based memristors for X_Mg_ = 0.11, 0.20, 0.27. Each pulse train consists of 4 pulses with 2 V amplitude, with different intervals between pulses. (c) Example of input spike trains generated from an audio waveform using a cochlear ear model, which are fed into the memristor reservoir. (d) Experimentally obtained confusion matrix for the RC system built with memristors of X_Mg_ = 0.11 under 100 ns input time‐step intervals, showing a high classification accuracy of 99.42%. (e) Experimentally obtained confusion matrix representing the classification outputs from the RC hardware system using ESOs with X_Mg_ =  0.11 for the 100 ns time interval test. Reproduced with permission [216]. Copyright 2024, The Authors, published by Springer Nature under a Creative Commons Attribution 4.0 International License. (f–j) Dynamic memristor‐based RC system for spoken‐digit recognition. (f) Schematic structure of the Ti/TiO_x_/Al_2_O_3_/TaO_y_/Pt memristor. (g) The typical audio waveform of the digit 9 transformed into the cochlear spectrum. (h) Input voltage signal after time multiplexing processing and memristor response. (i) Predicted output of RC systems based on memristors with correct output. (j) The relationship between word error rate and mask length, where the total database size remains constant at 400. Reproduced with permission [36]. Copyright 2021, The Authors, published by Springer Nature under a Creative Commons Attribution 4.0 International License.

For instance, Yoo et al. demonstrated a high‐performance spoken‐digit recognition RC system based on entropy‐stabilized oxide (ESO) memristors featuring tunable internal time constants (Figure 9a) [216]. The RC system consists of an input layer, a reservoir comprising 64 ESO memristors, and a trainable readout layer. By adjusting the magnesium composition in the film, the internal relaxation time constant of the memristors can be precisely engineered from an average of 159–278 ns, allowing the reservoir dynamics to be matched to different input signal timescales (Figure 9b). In this system, audio signals from the Audio‐MNIST dataset are first converted into 64‐channel spike trains via a cochlear ear model (Figure 9c). These spikes are fed into the corresponding ESO memristors, and the conductance state of each memristor is sampled at specific intervals to form virtual nodes, collectively creating a high‐dimensional reservoir state. Experimentally, the RC system constructed with memristors having the shortest relaxation time (X_Mg_ = 0.11) achieved a recognition accuracy of 99.42% when processing inputs with a 100 ns time‐step interval (Figure 9d). Crucially, the system's performance for inputs with different temporal characteristics can be optimized by selecting memristors with appropriate internal time constants. For instance, when processing slower inputs with longer time‐step intervals (250 and 400 ns), memristors with correspondingly longer relaxation times (X_Mg_ = 0.20 and 0.27) yielded superior accuracy (Figure 9e). This work highlights the significant advantage of RC systems for specific temporal tasks by engineering the fundamental physical properties, such as the time constant, of the memristive devices.

The richness of reservoir states is a key factor influencing the performance of RC systems. Traditional approaches often depend on the inherent variations among devices to generate diverse reservoir states. However, once the devices are fabricated, dynamically adjusting these states to meet specific task requirements becomes challenging, as the richness of these states is fixed. In 2021, Zhong and colleagues proposed an RC system based on a Ti/TiO_x_/TaO_y_/Pt memristor (Figure 9f), which utilizes controllable time‐multiplexing to generate rich and tunable reservoir states [36]. This allows not only changing the diversity of reservoir states but also regulating the system's feedback strength by flexibly adjusting parameters related to time multiplexing.

Specifically, the original audio waveform is first transformed into a 64‐channel spectrogram using the Lyon passive ear model (Figure 9g). Input voltage signals with time‐delay characteristics are obtained after mask operation. Then, these signals are fed into parallel memristors, which excite distinct virtual nodes (Figure 9h). These nodes are subsequently sent to a linear readout network to achieve accurate spoken‐digit recognition. Figure 9i shows a comparison between predicted and correct digits obtained from an RC system based on memristors. When the mask length is 10, and the number of parallel devices is 40, the word recognition rate reaches 99.6%. Furthermore, the error rate is a function of mask length. With the total device size fixed at 400, the average word error rate achieves its minimum value when the mask length is 10 (Figure 9j).

#### 3.1.3 Trajectory Recognition

The preceding sections demonstrate the capability of memristive RC systems to process static images and sequential acoustic signals. In addition, many real‐world intelligent tasks—such as motion tracking and gesture interpretation—involve recognizing dynamic trajectories, i.e., spatiotemporal patterns that evolve over time. This presents a more complex challenge, requiring the RC system to extract temporal features with high efficiency and low latency, often under resource‐constrained hardware conditions. A promising approach to address this challenge lies in the full hardware integration of RC, where both the reservoir and the trainable readout layer are implemented using memristive devices.

**FIGURE 10:**
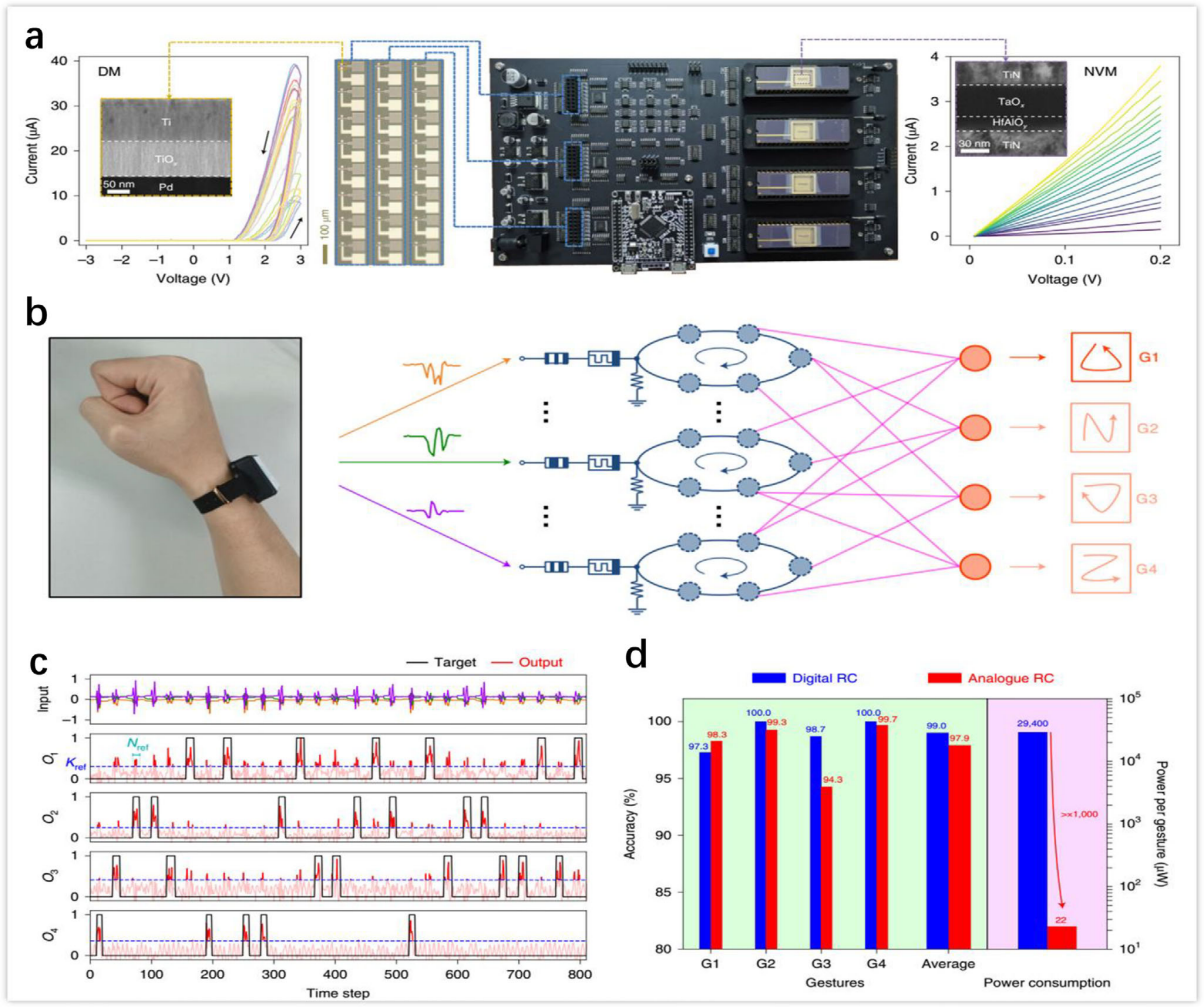
Hardware‐integrated RC system for dynamic gesture recognition. (a) Device characteristics of memristors and the architecture of the RC hardware system. (b) Flowchart of RC system operations in dynamic gesture recognition. (c) Demonstration of gesture signal input and RC system output signals. (d) Comparison of classification accuracy and power consumption between digital and fully analog RC systems. Reproduced with permission [41]. Copyright 2022, Springer Nature.

For example, Zhong et al. demonstrated an all‐analog RC system utilizing two types of memristors: volatile memristors form the physical reservoir, while non‐volatile memristor arrays serve as the readout layer (Figure 10a) [41]. The authors showcased the robust performance of their all‐analog memristive RC system in dynamic gesture recognition tasks. The study acquired four distinct dynamic gesture signals from the acceleration sensor (Figure 10b). Input signals underwent masked processing across 24 dynamic memristors, generating reservoir states that are fed into four non‐volatile resistive array units for weight training. During testing, the system processed mixed input signals encompassing all four gesture categories in real time. Four readout channels output recognition signals corresponding to different gestures. By setting two reference values (K_ref_ and N_ref_), the system ultimately achieves high‐accuracy gesture classification (Figure 10c). Experimental results demonstrate that this fully analog RC system achieves an accuracy rate of 97.9%. Compared to fully digital RC systems, the all‐analog RC system exhibits only a 1.1% accuracy loss while achieving a 99.9% reduction in power consumption (Figure 10d). This work powerfully demonstrates the potential of all‐analog memristive RC systems for complex spatiotemporal signals, providing a viable technical pathway toward realizing low‐power, real‐time intelligent computing.

**FIGURE 11:**
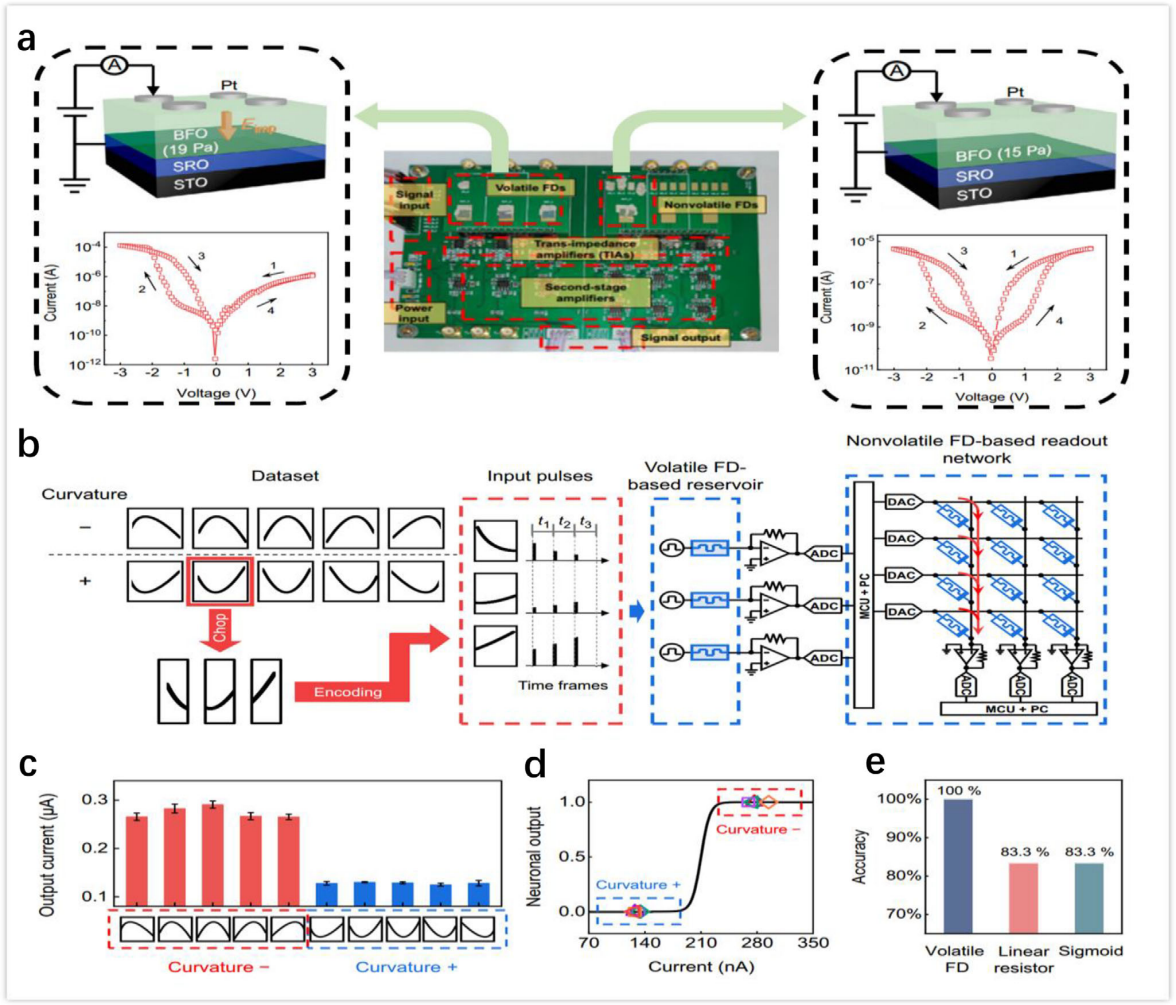
All‐ferroelectric RC system for curvature discrimination task. (a) System architecture of the all‐ferroelectric RC. The volatile ferroelectric diodes (FDs) form the reservoir, while the nonvolatile FDs constitute the readout network, as distinguished by their respective *I*–*V* scanning curves. (b) Implementation flow for curvature discrimination. Input curves are first segmented and converted into multi‐timeframe pulse trains, which are then processed by a volatile FDs reservoir. The resultant reservoir states are finally classified by a nonvolatile FDs‐based readout network. (c) The output current generated by the output neurons in the readout network and (d) the neuronal output through the sigmoid function. (e) Accuracy comparison of the all‐ferroelectric RC system and two controlled RC systems on the test set (36 curves). Reproduced with permission [37]. Copyright 2023, The Authors, published by Springer Nature under a Creative Commons Attribution 4.0 International License.

Furthermore, ferroelectric synapses have emerged as a promising class of neuromorphic devices and have garnered considerable attention in RC in recent years [116, 215, 220, 224, 231, 232, 233, 234, 235, 236]. Leveraging the polarization reversal properties of ferroelectric materials, they offer advantages such as low power consumption and high speed, demonstrating immense application potential in fields like neuromorphic computing and compute‐in‐memory technologies. Chen and colleagues developed a fully ferroelectric RC system using both volatile and non‐volatile ferroelectric memristors and evaluated its performance on a curvature discrimination task (Figure 11a) [37].

The study utilizes a dataset of 138 curves (half with positive curvature and half with negative), divided into 102 for training and 36 for testing. Each curve is segmented and converted into a pulse sequence across three time frames, which is then fed into the reservoir composed of three volatile ferroelectric diodes. These devices utilize their nonlinearity and short‐term memory to map the temporal information into a high‐dimensional space. The reservoir states are captured by reading the current response of the diodes after each pulse. This signal is processed by the readout network consisting of four non‐volatile ferroelectric diodes, which performs linear weighting followed by a Sigmoid activation function to produce classification output approaching 0 for positive curvature and 1 for negative curvature (Figure 11b). Experimental results demonstrate significant differences in network outputs between positively curved and negatively curved curves (Figure 11c,d). Furthermore, the authors compared the classification accuracy of three RC systems (fully ferroelectric RC system, linear resistor, and sigmoid function) across 36 test curves, with the fully ferroelectric RC system achieving 100% accuracy and successfully demonstrating its application potential (Figure 11e).

### 3.2 Sequence Prediction

Nonlinear time‐series forecasting is considered one of the key areas in the study of RC systems, which involves establishing nonlinear relationships to describe an unknown system based on a set of observed time‐series data without prior knowledge of the underlying mathematical model [125]. Traditional time‐series forecasting methods usually rely on explicit parametric models. Although these methods incorporate nonlinear terms, they are constrained by inherent limitations of the model and thus struggle to fully capture highly complex nonlinear relationships, particularly chaotic systems. In contrast, RC does not directly fit equations but instead utilizes a fixed, randomly initialized reservoir as a complex dynamic system to nonlinearly map time‐series data into a high‐dimensional space [237]. This mechanism efficiently extracts rich features from raw data and enables predictions using only a simple linear output layer. This architecture confers two advantages for RC: (i) the ability to approximate complex dynamics, and (ii) extremely low training costs, making it a powerful tool for analyzing nonlinear time‐series.

**FIGURE 12:**
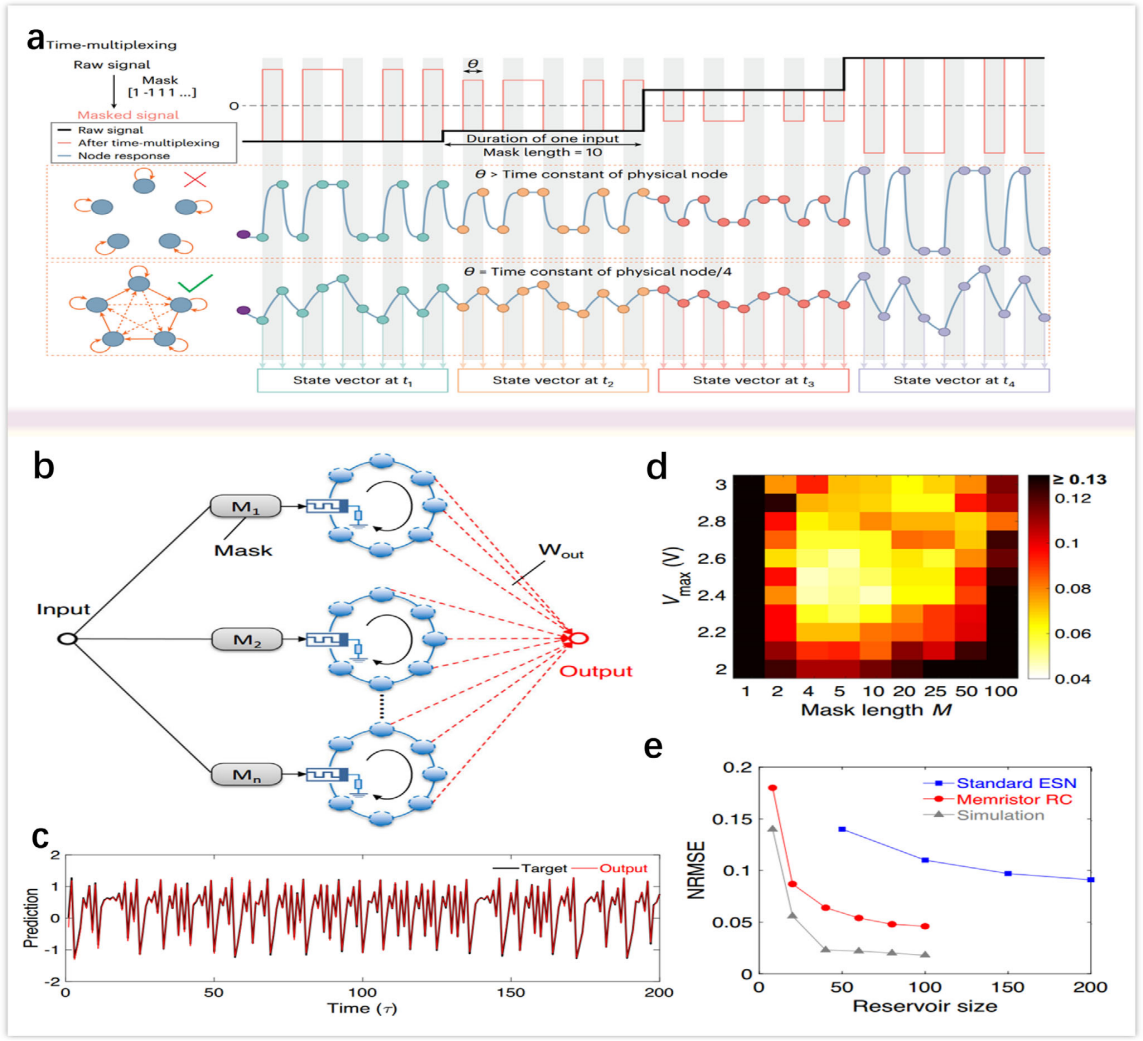
Demonstration of Hénon map prediction in RC system with mask. (a) The input signal employs a time‐multiplexing operation with a mask length of 10. Reproduced with permission [26]. Copyright 2024, Springer Nature. (b) Schematic of a parallel RC system and (c) prediction results based on dynamic memristors, where each memristor has a unique mask sequence. (d) 2‐D display of predicted results under different test parameters. (e) The relationship between NRMSE and reservoir size in different RC systems. Reproduced with permission [36]. Copyright 2021, The Authors, published by Springer Nature under a Creative Commons Attribution 4.0 International License.

Time multiplexing is a commonly used technique for enhancing reservoir state richness in sequence prediction. Wu's team systematically detailed the specific implementation steps of this method [26]. As shown in Figure 12a, when the mask length is 10, the duration of each discrete data point is divided into ten equal units. The value of each unit is randomly multiplied by ‐1 or 1, generating a mask vector that remains constant throughout the entire dataset. To stimulate complex dynamics, the duration (θ) of each unit is typically set to approximately one‐quarter of the physical node time constant. The virtual nodes generated by each unit are serially combined into a state vector. Here, careful selection of the unit duration θ and mask length is essential: if θ is too long, node responses may saturate prematurely, leading to insufficient state diversity; if θ is too short, nodes operate only within a narrow linear range, resulting in reduced response amplitudes. These scenarios demonstrate that the richness of the virtual node states is not arbitrary but is governed and constrained by the intrinsic dynamic characteristics of the memristor, specifically its relaxation time constant. Therefore, the time constant must be adjusted based on the required signal spacing and number of virtual nodes. The team proposed a parallel dynamic memristor RC system based on controllable masking techniques (Figure 12b), where key parameters—including state richness, feedback strength, and input scaling—can be adjusted by modifying the mask length and input signal range [36]. The system achieved a low normalized root mean square error (NRMSE) of 0.046 in time series prediction for the Hénon map (Figure 12c, d), outperforming most existing hardware‐based reservoir computing systems and even surpassing software‐based approaches for Hénon map prediction tasks (Figure 12e). This superior prediction performance can be attributed to the effective transformation of temporal input data into a high‐dimensional feature space. This transformation is facilitated by the memristors' nonlinear relaxation dynamics, which serve as the physical engine for time‐multiplexing. Thus, specific device dynamics are directly translated into a computational advantage for time‐series forecasting.

**FIGURE 13:**
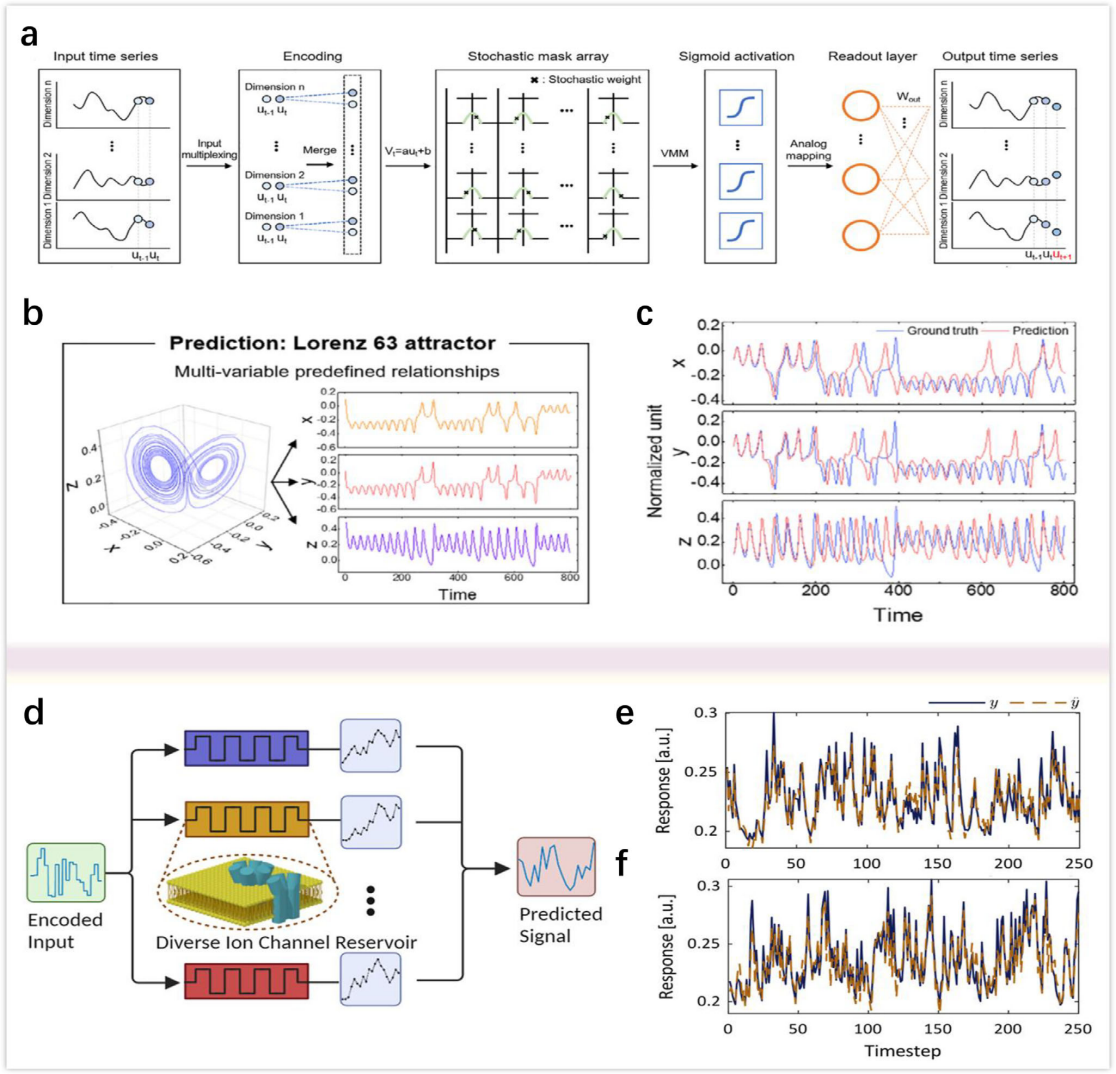
(a–c) Prediction of multivariate spatiotemporal time‐series data in RC system. (a) Schematic of proposed MESN operation. (b) Lorenz 63 data for prediction. (c) Short‐term prediction results for the Lorenz 63 benchmark. Reproduced with permission [64]. Copyright 2025, The Authors, published by Wiley‐VCH under the terms of the Creative Commons Attribution (CC BY) License. (d–f) Second order task prediction based on different memristors. (d) A diagram shows that the signal voltage waveform is sent to all ion‐channel‐based memristors, each exhibiting different temporal dynamics. (e) The training waveforms and (f) testing waveforms for the second‐order task without masking operations. Reproduced with permission [229]. Copyright 2024, American Chemical Society.

Current physical implementations of RC are primarily constrained by time‐delay architectures that lack intrinsic spatial data processing capability [43, 238, 239]. Memristive Echo State Networks (MESN) represent a multifunctional RC system capable of performing spatio‐temporal computations within a single device crossbar switch array. For example, Kim and colleagues achieved multivariate spatio‐temporal data prediction through the MESN system [64]. Figure 13a shows the multivariate spatio‐temporal data prediction of the MESN system. First, continuous multivariate time‐series data are discretized in both spatial and temporal dimensions: the spatial dimension corresponds to multiple variables, while the temporal dimension reflects the evolutionary interval. These discretized time‐series data are linearly mapped to voltage pulses in the memristor, forming the delayed input. Subsequently, the input is applied to a mask composed of randomly weighted crossbar array. The device current is output after being accumulated through multiplication. This current is converted into voltage and undergoes a Sigmoid‐like nonlinear activation implemented by the bistable memristor array, generating the final reservoir state for training. In the Lorenz 63 attractor prediction, the system normalizes the number of time series from the third‐order ordinary differential equations describing atmospheric convection (Figure 13b). The system consists of six delay embeddings and one bias term. It utilizes a 7 × 100 random masking array to map the input into a 100‐dimensional reservoir space for training the readout layer weights. In this work, MESN accurately tracks the dynamics of chaotic systems in short‐term predictions after 800 training time steps, achieving a low NRMSE of 0.077, which demonstrates that the RC system effectively captures complex dynamics (Figure 13c).

Implementing efficient time‐series forecasting with physical hardware‐based RC system is a challenging task. Traditional methods usually rely on complex masking techniques to perform time multiplexing on input signals, generating sufficient high‐dimensional virtual nodes from a limited number of physical devices. However, this approach introduces significant preprocessing overhead and constraints the richness of features derived from the same dynamic behavior. Armendarez et al. reported an ion‐channel‐based memristor that successfully achieves second‐order nonlinear dynamic system prediction without mask operations (Figure 13d) [229]. This RC system performs a simple linear encoding of the input signal and applies it to a parallel reservoir composed of memristors with five distinct Alamethicin concentrations. Due to their intrinsic dynamic parameters such as unique voltage thresholds and decay time constants, each memristor exhibits diverse and nonlinear conductance responses to the same input. Thus, higher prediction accuracy is achieved using only five physical devices, eliminating the need for masking (Figure 13e,f). This approach not only simplifies system architecture but also compensates for limited feature dimensionality by exploiting device‐level heterogeneity.

## 4 Optoelectronic Memristive RC

Although electrical memristors can perform complicated RC computational tasks based on the crossbar array structures, their effectiveness in processing temporal information is constrained by limited bandwidth and interconnect crosstalk to electrical signals. In contrast, optoelectronic memristors utilize optical signals, which are characterized by high bandwidth, minimum crosstalk, and low energy dissipation, to modulate device conductance. This not only enables ultrafast speeds, but also makes optical signals an ideal low‐energy medium for processing temporal information, facilitating the construction of dynamic systems required for RC [213, 220, 240, 241, 242, 243, 244, 245]. The intrinsic parallelism of optical inputs allows a single optoelectronic memristor to simultaneously receive and process multiple optical or optoelectronic hybrid signals, enhancing the efficiency and capability of the reservoir in mapping input signals to high‐dimensional states [96, 214, 238]. Optoelectronic RC also enables direct integration with in‐sensor and multi‐modal architectures, combining sensing and computing into one system. This integration supports early‐stage signal processing at the sensor level, thereby reducing data transfer requirements as well as overall power consumption and latency. Depending on the variety of processing tasks they address, optoelectronic RC systems are broadly categorized into single‐modal and multi‐modal architectures.

### 4.1 Single‐Modal RC

Single‐modal RC employs a fixed, randomly connected reservoir to process complex dynamics within a single date modality. Owing to its robust time‐series processing capability, it has been successfully applied in fields requiring time‐related characteristics within individual signals. Compared to electrical signals, which can only encode information by regulating limited parameters such as pulse amplitude [219] and frequency [220], optical signals inherently provide wavelength selectivity [246] and highly parallel input [238, 247].These properties allow optoelectronic memristors to multiplex multiple information streams using optical pulses of different wavelengths. The parallel transmission characteristics of light facilitate the synchronous input of multi‐channel optical signals within the same spatiotemporal dimension, significantly expanding the application of optical‐input RC.

**FIGURE 14:**
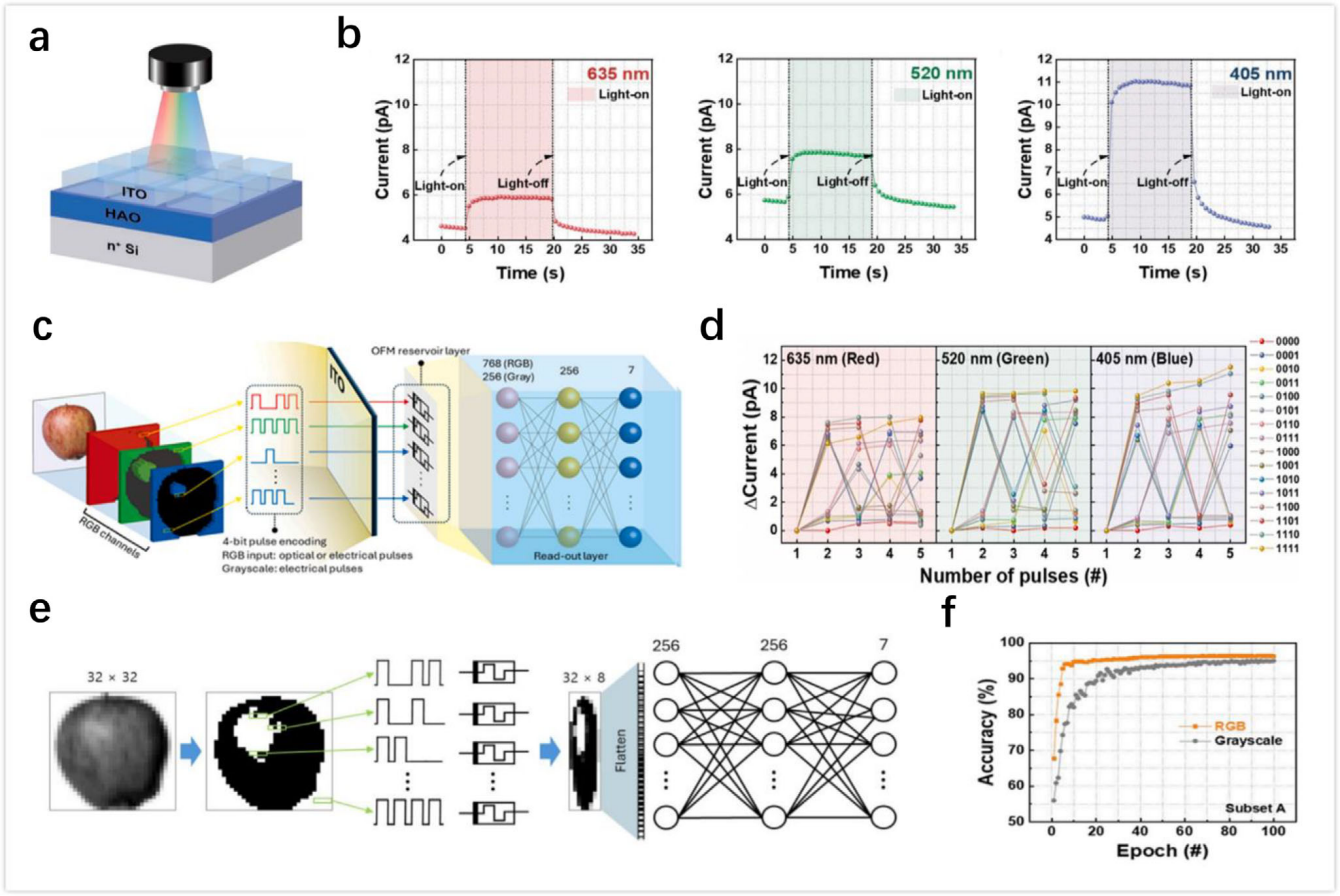
Memristive RC system for static image classification. (a) Schematic structure of the HfAlO_x_‐based memristor. (b) Current responses to optical stimulation with different wavelengths of light. (c) Schematic of optical input RC system for RGB image classification. (d) Current responses to 4‐bit optical stimulus measured at different wavelengths. (e) Schematic of the electrical input RC system for grayscale image classification. (f) Recognition accuracy for the Fruits‐360 dataset with color and grayscale image classification. Reproduced with permission [246]. Copyright 2025, Elsevier.

Furthermore, owing to their distinct dynamic responses to different light wavelengths, optoelectronic memristors enable RC systems to efficiently process color visual information. For example, Park et al. developed an RC system utilizing an optical ferroelectric memristor (OFM) to achieve high‐accuracy color image recognition (Figure 14a) [246]. This device exhibits distinct light current responses at different wavelengths, providing a solid hardware basis for color discrimination (Figure 14b).

In this system, the 32 × 32 pixel red‐green‐blue (RGB) image is first preprocessed by binarizing the pixel values in each color channel and encoding four pixels per row as a 4‐bit pulse sequence. Specifically, the red, green, and blue channels correspond to optical pulses at wavelengths of 635 nm (0.22 mW/cm^2^), 520 nm (0.289 mW/cm^2^), and 405 nm (0.283 mW/cm^2^), respectively. The optical pulses excite oxygen vacancy ionization in the ferroelectric layer through transparent ITO electrodes, generating a wavelength‐dependent optical response (Figure 14c). The nonlinear dynamic behaviors of the OFM device are utilized to convert optical pulse sequences into high‐dimensional state vectors of 768 dimensions (3 × 32 × 8) (Figure 14d). Finally, significant results are achieved in the fruit recognition task on the Fruits‐360 dataset through a 768 × 256 × 7 multi‐layer perceptron architecture. Before being fed into the reservoir, the dataset was first converted to grayscale and then encoded using electrical pulse encoding. Unlike three‐channel RGB images, grayscale images contain only a single channel, reducing the input node dimension to one‐third of the original (Figure 14e). This RC system demonstrates a recognition accuracy of 97.99% for samples exhibiting distinct color feature differences, representing an approximately 10% improvement over grayscale image precision (Figure 14f). Furthermore, to highlight the energy efficiency advantages of the optical input RC, a quantitative comparison of energy consumption is conducted for the same encoding pattern. These results show that electrical pulse encoding requires 1.61 × 10^−4^ J, whereas optical pulse encoding consumes only 5.75 × 10^−6^ J, demonstrating that optical encoding is approximately 27 times more energy‐efficient.

**FIGURE 15:**
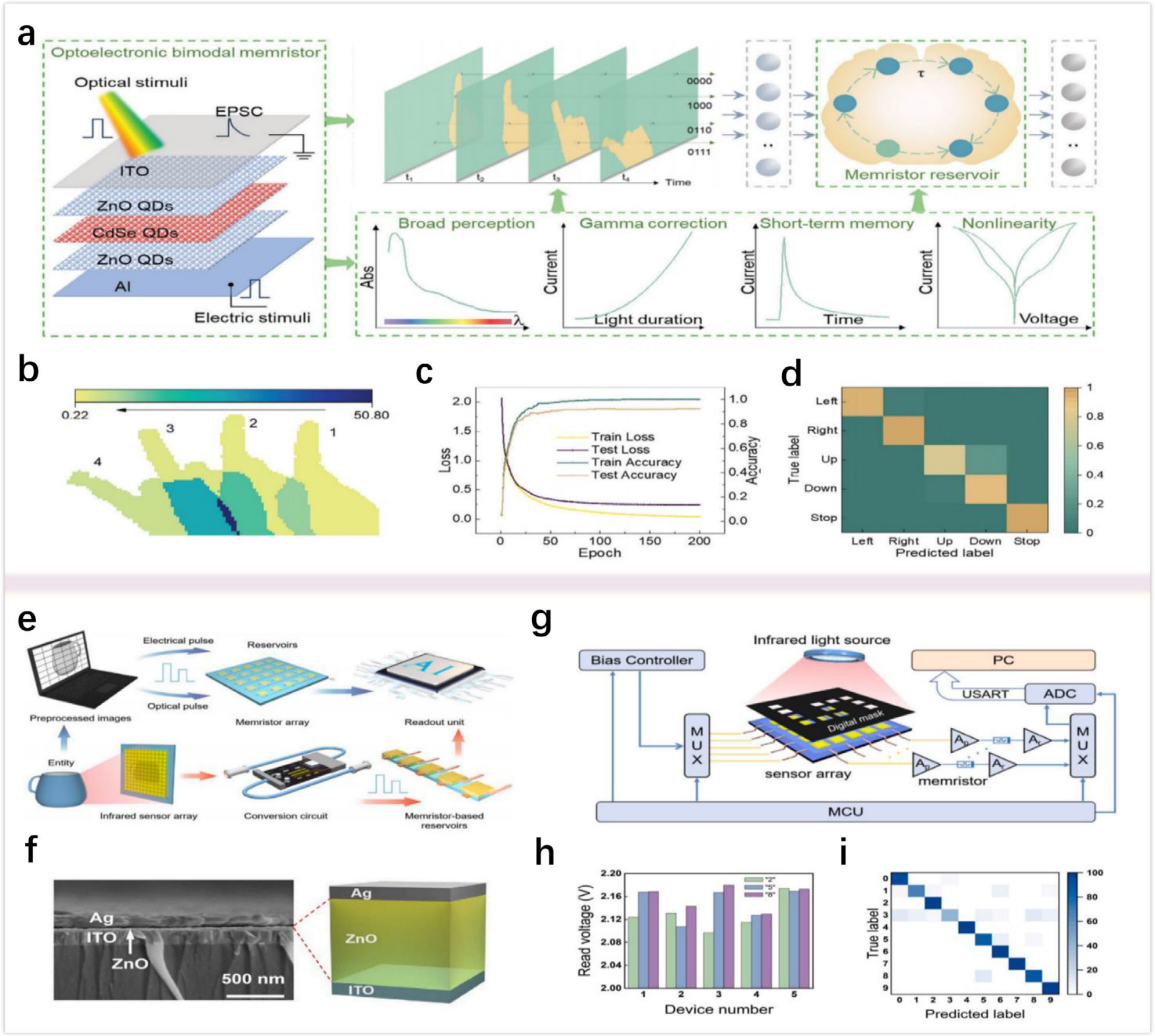
(a–d) Memristive RC system for dynamic image recognition. (a) Schematic of the FQDOM RC system for dynamic gesture recognition. The bottom‐right panel illustrates the computational properties of the device. It provides a schematic representation of its short‐term memory and nonlinear dynamic response to both optical and electrical stimuli. Together, these properties form the fundamental requirements for implementing a physical reservoir. (b) The reservoir state in the device under gesture motion conditions. (c) Accuracy and loss of dynamic gesture recognition with the FQDOM reservoir system, and (d) confusion matrix of the five gestures. Reproduced with permission [251]. Copyright 2025, Wiley‐VCH. (e) Comparison of two technical routes for realizing artificial vision based on a memristor RC system. The traditional route first converts the target into a preprocessed image, which is subsequently encoded as electrical or optical pulse sequences and fed into the reservoir for feature extraction. The route in this work directly generates a pulse stream containing image information from the sensing array, which is then delivered to the memristor reservoir for recognition, bypassing imaging steps. (f–i) The RC system is used to realize entity target recognition. (f) Schematic illustration of the Ag/ZnO_x_/ITO device. (g) Schematic of the RC system for entity target recognition. (h) Reservoir states corresponding to the three examples. (i) Confusion matrix showing the entity target recognition results of the memristor‐based RC system. Reproduced with permission [254]. Copyright 2025, Elsevier.

The essence of the optical input RC system is in extracting spatio‐temporal features embedded within complex time‐series through dynamic systems, enabling the RC system to process not only static but also dynamic visual information [248, 249, 250]. Dynamic gesture recognition, a typical sequential signal processing task, is critical for capturing spatio‐temporal features within continuous gesture sequences. Full quantum dot optoelectronic memristors (FQDOMs) were employed to construct a RC system, which demonstrated high‐precision recognition​ of five distinct dynamic gestures, as reported by Lin et al. (Figure 15a) [251]. This demonstrates the effectiveness of the RC system in processing dynamic stream data. Specifically, each gesture is converted into four frames of images, with the pixels of each frame input into the FQDOM reservoir as optical pulses. Leveraging STM and nonlinear response mechanisms, it maps low‐dimensional gesture features to a high‐dimensional state space. Notably, the optical pulse from the final frame stands out the most within the reservoir. The overlapping regions across multiple frames generate stronger output signals due to consecutive pulses, highlighting the trajectory of the gesture (Figure 15b). The study employs a single‐layer CNN as the readout layer, achieving 100% training accuracy and 92.59% test accuracy within only 200 training cycles (Figure 15c,d).

The typical architecture of single‐model RC based on memristors can be referenced in the process indicated by the blue arrows in Figure 15e. In particular, reservoir structures of optoelectronic devices typically require an analog‐to‐digital conversion module to drive the light source [85]. Image information is then transmitted into the reservoir through optical signals, which increases the complexity of the system. Furthermore, most studies utilize preprocessed images as input signals for RC systems. This way lacks completeness because it is necessary to identify entities rather than intermediate images for the hardware in artificial visual systems [249, 252, 253]. Wu and colleagues achieved entity target recognition based on an NIR sensing RC system [254]. Their design directly projects NIR light through a digital mask onto a PbS quantum dot sensor array. A bias controller generates specific voltage sequences to drive the sensor, producing a temporal response signal. This signal changes the conductance state of a reservoir composed of five ZnO memristors, thereby nonlinearly mapping the temporal signal into a high‐dimensional space. The voltage output from the reservoir is acquired through functional circuits and transmitted to the computer. Finally, the computer utilizes the pre‐trained CNN to classify the reservoir state and output the recognition result (Figure 15f,g). Figure 15h displays the reservoir states measured by the RC system for three categories of samples: the digits “2”, “5”, and “8”. This demonstrates that different categories of numerical inputs trigger distinguishable dynamic response patterns within the reservoir. Based on the confusion matrix, the overall recognition accuracy of the system on the test set reached 88.7%. This result demonstrates the effectiveness and feasibility of the NIR sensing RC system for entity target recognition tasks (Figure 15i). Notably, although this RC system does not employ optoelectronic memristors to construct a true optical input architecture, its significance lies in realizing a RC solution that transitions directly from physical signal perception to intelligent classification. This system design offers insights into the future development of more highly integrated sensor‐based computing systems.

**FIGURE 16:**
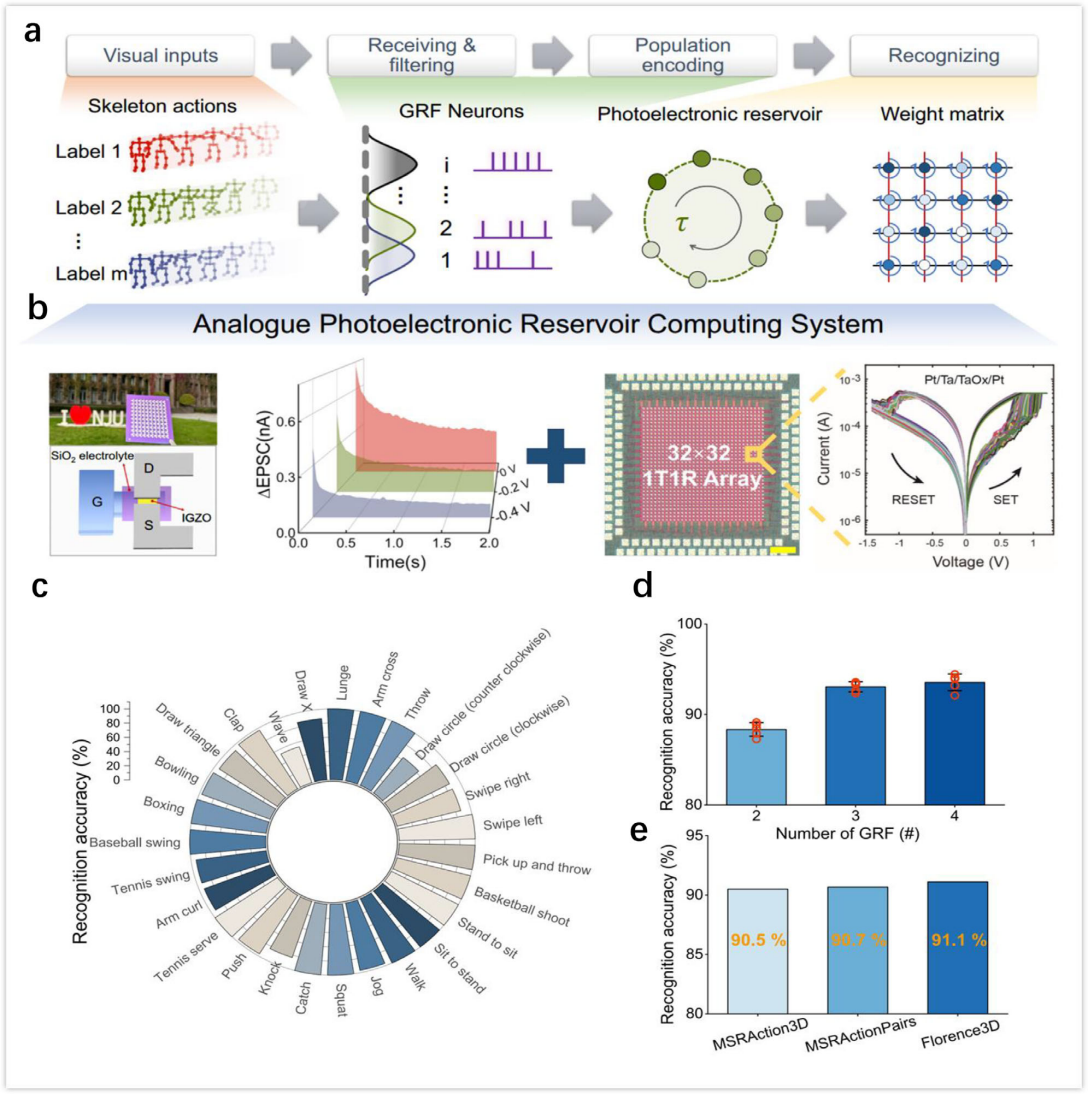
A bioinspired in‐material analog photoelectronic RC system. (a) Schematic diagram of the RC system for skeleton actions. (b) Architectural diagram of an analog optoelectronic RC system. IGZO optoelectronic transistors serve as the reservoir, while the 1T1R array is used for readout network weight updates. (c) Recognition results of bioinspired RC on the UTD‐MHAD dataset. (d) The recognition accuracies of the UTD‐MHAD dataset standard test with different numbers of GRF, and (e) different validation datasets. Reproduced with permission [57]. Copyright 2025, The Authors, published by Springer Nature under a Creative Commons Attribution 4.0 International License.

The RC vision system, which integrates optoelectronic synaptic devices with memristor‐based readout layers, effectively mimics human visual processing. This is accomplished by leveraging the intrinsic dynamics of optoelectronic synapses to map visual inputs into a high‐dimensional space for feature extraction, while the memristor crossbar array provides hardware acceleration for efficient recognition of dynamic information. For instance, a RC vision system was proposed based on optoelectronic synapse and memristor for efficient human motion recognition tasks (Figure 16a) [57]. Inspired by the information processing mechanisms of the human visual system, this system integrates IGZO optoelectronic synapse transistors as the reservoir layer and TaO_x_ memristor arrays as the output layer, constructing a dynamic visual processing platform with high energy efficiency and real‐time processing capabilities (Figure 16b). The system takes 3D human skeleton sequences as input and converts motion data into pulse sequences through a Gaussian Receptive Field (GRF) encoding mechanism, eliminating the need for complex feature extraction steps in traditional algorithms. It achieves a recognition accuracy of 93.58% on the UTD‐MHAD dataset, with 100% recognition for 14 action categories, demonstrating its capability to process complex motions (Figure 16c). Optimal recognition performance is achieved using three GRF neurons, consistent with reservoir memory capacity evaluations (Figure 16d). Ultimately, the system demonstrated recognition accuracy exceeding 90% across multiple datasets, demonstrating strong generalization capabilities and practical applicability (Figure 16e).

### 4.2 Multi‐Modal RC

A multi‐modal RC system refers to an architecture that integrates heterogeneous data streams—originating from distinct modalities or physical domains—into a unified RC framework for synchronous and collaborative processing. By means of a shared reservoir, such a system nonlinearly projects input signals from various modalities into a common dynamic feature space, thereby enabling the extraction and fusion of cross‐modal information. In contrast, single‐modal RC is limited to processing a single data stream, and its performance heavily depends on the completeness and quality of the available information. Multi‐modal RC, however, can tackle more complex temporal tasks by leveraging cross‐validation and cooperative characterization across different modalities. For instance, in a multi‐modal RC system implemented with optoelectronic memristors, the synergistic incorporation of both electrical and optical stimuli combines the advantages of the two excitation modes, endowing the memristor with richer and more tunable nonlinear response characteristics [54, 95, 96, 214, 238, 255, 256]. Electrical signals can modulate the conductance states of the memristor or provide essential electrical driving forces. Optical signals, known for their high bandwidth and low crosstalk, can also induce memristor conductance changes and trigger nonlinear dynamic behaviors that differ from those governed purely by electrical pathways [78, 83]. The coordinated input from both modalities not only expands the state‐space dimensionality of the memristor but also strengthens its nonlinear mapping ability through electro‐optical interactions. Consequently, a single device can exhibit more complex and controllable dynamic responses over a wider input range, ultimately enhancing the feature extraction capacity and prediction accuracy of the RC system in processing temporal signals.

**FIGURE 17:**
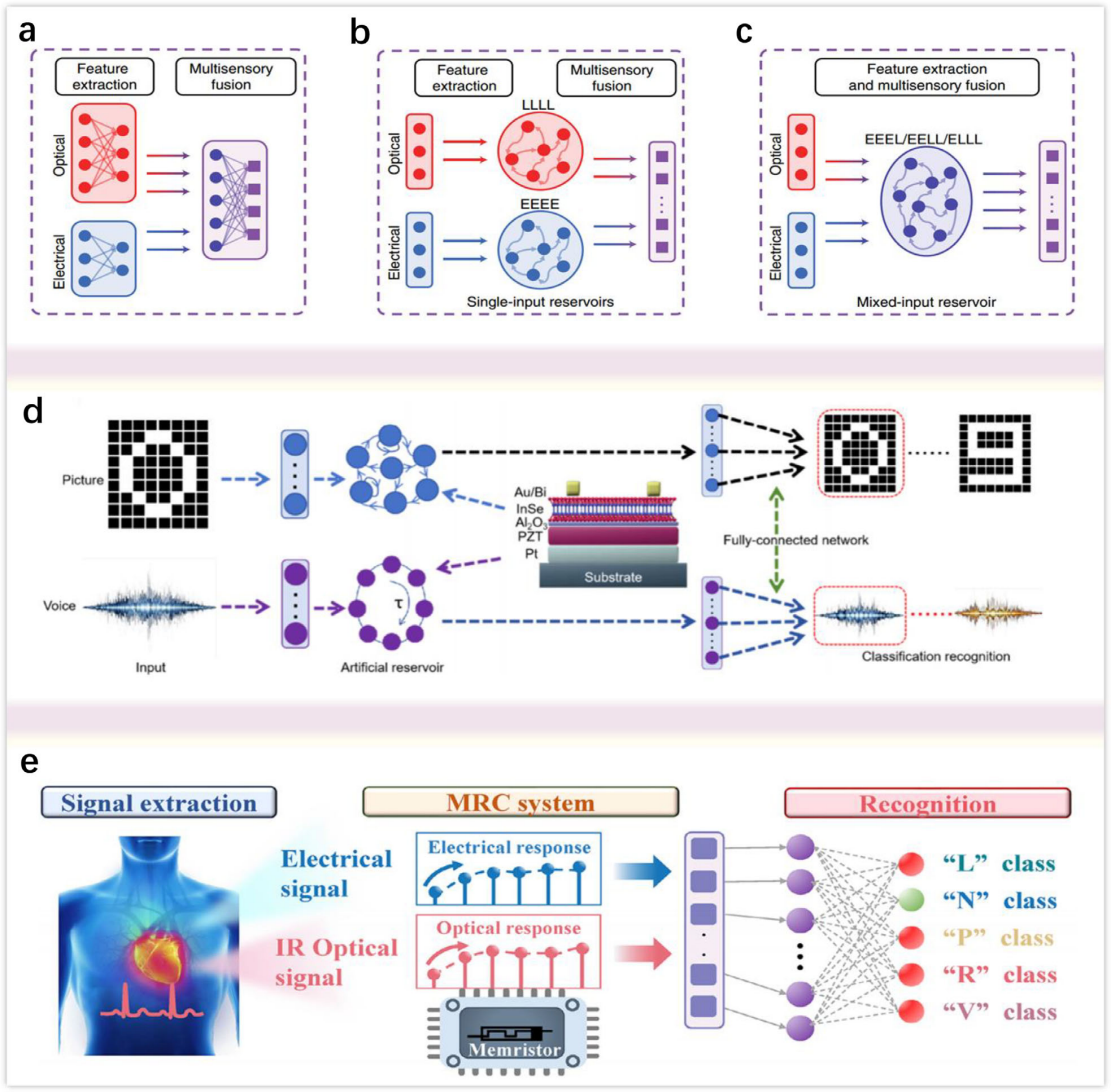
Schematic of the multi‐modal RC system. (a–c) Schematics of different multisensory fusion architectures. Reproduced with permission [55]. Copyright 2022, Springer Nature. (d) Schematic of the single‐input RC system for image and voice. Number‐image and voice information processed by the InSe based reservior, respectively, and subsequently transimitted to a fully connected neural network for recognition. Reproduced with permission [255]. Copyright 2025, American Chemical Society. (e) Schematic of the mixed‐input RC system for ECG pattern recognition. It contains the signal extraction, memristor reservoir, and the readout neural network. Reproduced with permission [96]. Copyright 2025, The Authors, published by Wiley‐VCH under the terms of the Creative Commons Attribution (CC BY) License.

Conventional memristive RC systems are limited by inherently fixed timescales and nonlinearities, as their relaxation dynamics and physical mechanisms are difficult to modulate. This inherent constraint restricts their capacity to process complex, multimodal information effectively. To overcome these limitations, Liu et al. demonstrated a multimodal physical RC system based on α‑In_2_Se_3_ synaptic devices [55]. Leveraging the ferroelectric and optoelectronic properties of α‑In_2_Se_3_, a reservoir with tunable nonlinear dynamics is created for multimodal signal processing. Rather than separately extracting features from each modality for later fusion via an ANN (Figure 17a), their work employed more efficient physical RC approaches. The first approach adopts a single‑input reservoir architecture, where dedicated reservoir layers, each responsive to a specific type of input signal (e.g., electrical or optical), perform independent feature extraction. The resulting features are subsequently combined and classified in the readout layer (Figure 17b). The second, more integrated approach is the mixed‑input reservoir architecture, in which a single reservoir simultaneously responds to multiple physical stimuli, thereby enabling multimodal fusion directly within the physical feature‑extraction process (Figure 17c). To validate the superiority of the mixed‑input reservoir, a multimodal handwritten digit recognition task is designed: the left half of each image is encoded exclusively into electrical pulses, while the right half is encoded solely into optical pulses. Experimental results show that single‑input reservoirs, which can only process partial information, achieve an accuracy of 73%. In contrast, the mixed‑input reservoir architecture attain a significantly higher accuracy of 85%. This performance gain stems from the tight coupling between ferroelectric and optoelectronic processes within the α‑In_2_Se_3_ device, which enables deep physical‑level fusion of the nonlinear dynamic responses triggered by electrical and optical inputs.

In multi‐modal RC systems, the simulation of audiovisual perception constitutes a representative application paradigm. In such implementations, optical signals typically emulate visual information, whereas electrical signals are utilized to represent auditory inputs. This approach aligns with the intrinsic multi‐sensory processing mechanisms observed in biological systems. For instance, Chen et al. demonstrated a single‐input multi‐modal RC architecture based on In_2_Se_3_ synaptic devices, designed to mimic the integrative audiovisual perception mechanisms in humans [255]. The system achieves efficient multimodal recognition by separately extracting spatial information features from visual data and temporal information features from auditory data on the same device (Figure 17d). It features two distinct reservoir structures to process spatial and temporal signals, both demonstrating outstanding recognition performance. In spatial signal processing, the image pixel information is mapped into high‐dimensional features by inputting optical pulse sequences into In_2_Se_3_ devices, which excite the optical current response of the devices, achieving 100% accuracy in digital image recognition tasks. For temporal signal processing, the system employs a delayed RC architecture to process speech signals. The speech waveform undergoes preprocessing through a masking matrix, converting it into a time‐multiplexed sequence of voltage pulses. These pulses generate virtual nodes upon input to the In_2_Se_3_ devices, with classification performed by the readout layer. This method achieves a 99% accuracy rate in speech recognition tasks, enabling highly efficient auditory signal processing. For single‐input reservoirs, these two signal channels remain isolated without direct interaction in the reservoir space. While this reduces the maximum reservoir space required by physical devices, it also means the system requires more hardware units to achieve the corresponding functionality.

Compared to a single‐input reservoir, a mixed‐input reservoir enables the parallel processing of multimodal signals within a same reservoir space, substantially lowering the hardware overhead of the memristor array. Nevertheless, this integrated approach places higher demands on the high‐dimensional transformation capability of RC devices, which should be enhanced to more accurately capture and represent the underlying interactions among multidimensional signals. Figure 17e presents a successful example of a multi‐modal fusion RC system based on Te optoelectronic memristors [96]. This system utilizes a single memristor to synchronously process two distinct modalities of information, including infrared light pulses and electrical pulses, thereby simulating the multi‐modal sensory integration capabilities of biological neural systems. It is employed for electrocardiogram (ECG) pattern recognition tasks. Five distinct types of heartbeat patterns are extracted from public datasets and the temporal waveforms of each pattern are converted into alternating sequences of electrical and optical pulses as hybrid inputs for the RC system. The short‐term plasticity of memristors allows for mapping the nonlinear dynamics of input signals into a high‐dimensional reservoir state space. By discretizing the continuous current response into virtual nodes, the system generates time‐series feature vectors, which are ultimately classified by a simple readout layer. Notably, by integrating information from both optical and electrical input, this RC system improves ECG pattern recognition accuracy to 95.7%, significantly exceeding the accuracy achieved through either single electrical input (86.3%) or optical input (81.0%). This result demonstrates the advantages of multisensory fusion in enhancing the accuracy of complex temporal information processing and its application in fields such as real‐time health monitoring.

## 5 Comparison of Different Memristive RC

Table 1 provides a comparative summary of the device structures, operational modes, and representative applications of both electrical and optoelectronic memristor‐based RC systems, illustrating the progressive advancement of memristive RC technology.

**TABLE 1:** Comparison of memristor‐based RC in representative studies.

Device type device structure		Mode	Input signal	Application	Accuracy	Year	Refs.
EM^a^	W/WO_x_/Pd/Au	single‐mode	Electrical	Handwritten digit recognition Time‐series forecasting	88.1% NMSE^c^:0.0036	2017	[65]
	Ti/TiO_x_/TaO_y_/Pt	single‐mode	Electrical	Waveform classification Spoken‐digit recognition Time‐series forecasting	NRMSE^d^: 0.14 99.6% NRMSE: 0.046	2021	[36]
	Ti/TiO_x_/Pd	single‐mode	Electrical	Arrhythmia detection Dynamic gesture recognition	96.6% 97.9%	2022	[41]
	Au/MAPbI_3_/ITO	single‐mode	Electrical	Image recognition	90.1%	2022	[48]
	Pt/BiFeO_3_/SrRuO_3_	single‐mode	Electrical	Curvature discrimination Digit recognition Waveform classification Time‐series forecasting	100% 91.7% NRMSE: 0.13 NRMSE: 0.017	2023	[37]
	TiN/Al‐HfO_2_/ZrO_2_/n^+^Si	single‐mode	Electrical	Handwritten digit recognition	95.83%	2024	[224]
	ITO/NiO_x_/WO_x_/Pt	single‐mode	Electrical	Handwritten digit recognition	96.2%	2024	[90]
	ITO/IGZO/TaN	single‐mode	Electrical	Handwritten digit recognition	94.1%	2024	[212]
	Pt/Ti/ESO/YBCO/MgO	single‐mode	Electrical	Spoken‐digit recognition	99.42%	2024	[216]
	ITO/CsCu_2_I_3_/Au	single‐mode	Electrical	Alphabet recognition	98.2%	2024	[257]
	ITO/ZrO_x_/TaN	single‐mode	Electrical	Handwritten digit recognition	96.3%	2024	[258]
	Ag/Pentacene/Ag	single‐mode	Electrical	Morse code recognition Handwritten digit recognition	100% 91.6%	2025	[223]
	Pt/TiO_x_/AlO_y_/Pt/Ti	single‐mode	Electrical	Dynamic gesture recognition	93.9%	2025	[79]
OM^b^	Au/Cr/PMMA:SnS/Cr/Au	single‐mode	Optical	Alphabet recognition Sentence recognition	100% 91%	2021	[259]
	Au/P(VDFTrFE)/Cs_2_AgBiBr_6_/ITO	single‐mode	Optical	Face classification	99.97%	2022	[220]
	Au/ZnO:N/IGZO/TiN	single‐mode	Optical	Handwritten digit recognition Action classification	90.45% 97.14%	2022	[248]
	Ti/a‐GaO_x_/Ti	single‐mode	Optical	Fingerprint recognition	100%	2022	[85]
	Au/PbS/Al_2_O_3_/Si/In‐Ga	single‐mode	Optical	Image recognition	96.5%	2024	[78]
	ITO/NiO_x_/Au	single‐mode	Optical	Handwritten digit recognition	90.88%	2024	[260]
	Si/SiO_2_/TiN/TiO_2_/NbO_x_/NiO/Ru	single‐mode	Optical	Image recognition	93%	2024	[243]
	ZnO QDs/CdSe QDs/ZnO QDs	single‐mode	Optical	Dynamic gesture recognition Image recognition	92.59% 95.16%	2025	[251]
	Pt/OMeTAD/PVK/SnO_2_/ITO	single‐mode	Optical	Handwritten digit recognition	97.94%	2025	[261]
	FTO/Carbon‐MoS_2_/Ag	single‐mode	Optical	Alphabet recognition	100%	2025	[244]
	Au/Pd/α‐In_2_Se_3_/Pd/Au	multi‐mode	Electrical Optical	Handwritten digit recognition QR code recognition Time‐series forecasting	86.1% 98.6% NRMSE: 0.105	2022	[55]
	FTO/TiO_2_/Au	multi‐mode	Electrical Optical	Handwritten digit recognition Spoken‐digit recognition	90% 94.67%	2024	[242]
	TiN/ZnO/TiN	multi‐mode	Electrical Optical	Handwritten digit recognition Action classification Dynamic image recognition	94.1% 99.4% 100%	2024	[83]
	InSe/Al_2_O_3_/PZT	multi‐mode	Electrical Optical	Time‐series forecasting Waveform classification Image recognition Speech recognition	NRMSE:0.0535 NRMSE:0.0873 100% 99%	2025	[255]
	Au/TiO_x_/ITO	multi‐mode	Electrical Optical	Arrhythmia detection Handwritten digit recognition	95.1% 97.3%	2025	[262]
	Au/2H‐MoTe_2_/Au	multi‐mode	Electrical Optical	Alphabet recognition	82.83%	2025	[256]

^a^ electrical memristor.

^b^ optoelectronic memristor.

^c^ normalized mean square error.

^d^ normalized root means square error.

A diverse range of material systems have been investigated for the development of memristive RC systems. Among these, oxide materials have emerged as the most widely used platform due to their excellent compatibility with existing CMOS processes, superior chemical and thermal stability, and diverse fabrication methods. In electrical memristors, the switching of conductance states, which is central to implementing RC, is typically achieved by controlling the morphology of conductive filaments based on oxygen vacancies within the oxide layer. However, the inherent stochasticity in filament formation and dissolution hampers the precise control of reservoir states. To enhance device stability, researchers utilize bilayer oxide heterostructures (e.g., TiO_x_/TaO_y_ [36], BiFeO_3_/SrRuO_3_ [37], NiO_x_/WO_x_ [90], Al‐HfO_2_/ZrO_2_ [224], and TiO_x_/AlO_y_ [79]) to confine the location of filaments rupture and reduce variability. Furthermore, the wide bandgap of many oxides confines their photoresponse to the ultraviolet spectrum, limiting their utility in visual RC systems. In order to broaden the spectral range of optoelectronic memristors, high‑photosensitivity materials such as perovskites and two‑dimensional materials have been investigated, leading to the development of high‑performance RC devices. Nevertheless, issues related to process compatibility and long‑term stability introduced by these new materials must be carefully considered.

In terms of functional applications, memristor‐based RC devices have been primarily demonstrated in conventional domains such as static image recognition, time‐series prediction, waveform classification, and speech recognition, with performance typically quantified by specific tasks. For electrical memristors, research focus remains on pattern recognition and sequence prediction tasks. In contrast, optoelectronic memristors are inherently suitable for visual information processing, including in‐sensor RC technology. Notably, interest is growing in applying memristive RC to dynamic visual processing, where synergistic electrical and optical modulation can enable efficient handling of temporally evolving visual inputs.

Furthermore, the table underscores recent progress in multi‐modal RC systems, particularly the integration of electrical and optical inputs to perform recognition tasks on multi‐dimensional temporal signals. This transition from single‐modal to multi‐modal architectures underscores the superior capability of memristive RC in addressing complex, real‐world information processing tasks. The significance of multi‐modal RC lies in its capacity to leverage complementary information across sensory domains, thereby enhancing robustness and accuracy in complex temporal tasks. By concurrently processing heterogeneous data streams within a unified dynamic framework, multi‐modal RC can mitigate uncertainties inherent in single‐modal systems, support cross‐modal validation, and extract richer feature representations. This integration enables more nuanced modeling of real‐world phenomena where inputs naturally span multiple physical modalities, ultimately advancing the development of adaptive, neuromorphic computing systems capable of a human‐like perceptual system.

## 6 Challenges and Prospects

Physical RC represents a significant neuromorphic computing paradigm inspired by the brain's information processing mechanisms. Its core concept involves utilizing a fixed reservoir with complex dynamic properties to nonlinearly map low‐dimensional temporal input signals into a high‐dimensional state space. This architecture simplifies the training of RNNs by requiring only a simple readout layer, offering an attractive solution for efficient time‐series signal processing.

Memristors are widely regarded as the fourth fundamental circuit element following resistors, capacitors, and inductors. Two inherent properties make them an ideal platform for physical RC: first, their resistance value depends on the “fading memory” characteristic of past current or voltage excitation, naturally meeting the requirement for STM capability in RC; second, their intrinsic nonlinear current‐voltage relationship provides the physical foundation for achieving high‐dimensional nonlinear mapping of input signals.

This review has traced the evolution of RC systems from conceptual proposal to physical implementation. By focusing on the dynamic mechanism of memristors, it highlights the unique advantages and recent progress of memristor‐based RC systems. RC systems utilizing memristors have exhibited outstanding performance across diverse applications in recent years, including static image recognition, speech recognition, dynamic recognition, and time‐series prediction. Additionally, the systems not only process traditional electrical pulse signals but also leverage the high bandwidth and low crosstalk advantages of optical signals, achieving efficient color image recognition and multisensory fusion. This signifies the transition of the physical RC from mechanism validation to a new phase of functional diversification. Although memristive physical RC has obtained remarkable achievements in device mechanisms, system architectures, and application exploration, several critical challenges remain to be addressed before large‐scale applications can be realized.

### 6.1 Fully Hardware‐Based Memritive RC System

Current studies on memristor‐based RC are primarily focused on device‐level proof of concept, rather than fully integrated hardware systems. In a typical architecture, volatile memristors with nonlinear dynamics serve as the reservoir for feature extraction, while the readout function for classification is commonly implemented using software‐based ANNs algorithms. Although the physical memristive reservoir can efficiently extract features, the iterative training involved in the fully connected layers of ANNs imposes a high computational burden on conventional hardware. In recent years, significant progress has been made in using memristor crossbar arrays to accelerate ANNs computation, highlighting the importance of developing fully hardware‐ centric memristive RC systems to further improve energy efficiency. Achieving full hardware integration requires co‑designing volatile memristors with rich short‑term dynamics as the reservoir and non‑volatile memristor crossbars for trainable analog readout layers, complemented by on‑chip learning circuits and memory‑based architectures. This integrated approach is essential to overcome the latency and energy limitations of current hybrid analog—digital systems [37, 86, 116].

Furthermore, analog‑to‑digital conversion in memristive RC often introduces substantial energy overhead. Developing fully analog memristive RC systems is therefore critical, despite potential trade‑offs in computational accuracy. For example, Zhong et al. demonstrated that a fully hardware‑based RC system operating entirely in the analog domain can reduce power consumption by three orders of magnitude compared to an all‑digital RC system, underscoring the energy advantage of fully analog architectures [41]. The integration of a complete hardware RC system involves efficient interconnection among the reservoir‐layer memristors, readout‑layer memristors, and peripheral control circuits. Consequently, designing compact memristive RC architectures to minimize hardware overhead represents a key research direction for developing high‑efficiency RC systems in the future.

### 6.2 Multi‐Modal RC System for Real‐Time Perception and Processing of Physical Signals

Most reported multi‐modal RC systems do not process physical signals directly from real‐world sensors. Instead, they typically rely on pre‐recorded datasets, where inputs are preprocessed and encoded as electrical or optical signals. This approach fails to demonstrate the true capacity of multi‐modal RC for direct perception of realistic, noisy, and dynamically varying physical stimuli [95, 255]. Moreover, in practical settings, complex signal interference and noise place greater demands on the reservoir's state spaces and the adaptability of its nonlinear dynamics. By neglecting real‐time sensory input, such systems cannot fully validate their performance under actual operating conditions.

Further challenges arise from compatibility issues between sensors and physical reservoirs. Sensors often output continuous analog signals that require analog‐to‐digital conversion to interface with the reservoir dynamics. This conversion not only introduces additional latency but also increases power consumption, undermining the efficiency advantages of RC. A promising direction to address these limitations is in‐sensor computing technology, where the sensing unit itself forms part of the reservoir. By employing memristive materials with rich nonlinear responses, volatile devices can be designed to both detect environmental stimuli and generate complex spatiotemporal dynamics concurrently. When target information is perceived, it is directly projected into a high‐dimensional state within the reservoir through the intrinsic material dynamics, bypassing digital preprocessing and achieving seamless integration of perception and computation.

However, most currently reported in‐sensor RC schemes have yet to achieve real‐time perception and processing of physical signals. Continued advances in optoelectronic neuromorphic devices nonetheless offer promising pathways for next‐generation in‐sensor RC. For example, Chen et al. developed an optoelectronic graded neuron array for in‐sensor motion perception [263]. Inspired by the insect visual system, each unit performs nonlinear temporal coupling of optical signals, directly mapping the spatiotemporal features of motion trajectories through the device's current response. The system attained 99.2% accuracy in motion‐direction recognition, demonstrating its ability to perceive and process real‐world visual signals in real‐time. Although this work did not implement a complete RC architecture, the operational mechanism of the graded neurons closely mimics that of RC devices, thereby providing a valuable reference for future in‐sensor RC designs.

### 6.3 New Bipolar and Parallel Encoding Strategies

The distribution state of reservoir nodes directly determines the ability of the system to distinguish input signals. Excessively overlapping reservoir distributions will increase the challenge of high‐precision targets recognition in RC systems. However, traditional optoelectronic RC devices are typically limited by their unipolar light response. This unipolar coding response constrains the dynamic range and nonlinearity of the reservoir, leading to reduced diversity in internal reservoir states. Strong input signals may cause device failure and reduce the performance of temporal recognition tasks.

Although researchers have introduced hybrid photoelectric encoding strategies to enhance RC accuracy, these approaches also increase system complexity and introduce additional energy consumption [83, 238]. In addition to single‐source signal processing, advanced edge intelligence also requires the capability to efficiently process multi‐source information. However, existing physical RC systems typically process single‐source signals through serial encoding. Dealing with multi‐source tasks typically requires multiple independent reserves to operate in parallel.

In contrast, parallel encoding promotes efficient multi‐source information processing by combining feature extraction with information fusion and minimizing resource consumption. Achieving parallel encoding of multi‐source signals within the same reservoir device remains challenging. It requires a closely coupled physical mechanism to enable direct interaction among different signals. Therefore, there remains an urgent need for a single, simple device platform that inherently provides rich nonlinear dynamics for high‐precision reservoir computing and natively supports the efficient processing of multi‐source signals without external control complexity. For example, AOC memristors [195, 264, 265] and similar bipolar optoelectronic devices [266, 267] exhibit bidirectional optical responses, allowing reversible modulation of conductance states by adjusting the wavelength or power density of incident light. This capability is expected to expand the effective state space of the reservoir, thereby enhancing RC accuracy and robustness. Additionally, leveraging the high bandwidth of optical signals and the bipolar optical response of AOC devices makes it possible to achieve parallel encoding in physical reservoir computing.

### 6.4 Reliable and Stable Large‐Scale Fabrication of Memristive Devices

The transition from lab‐scale proof‐of‐concept to industrially viable, large‐scale integration of memristive RC systems hinges on overcoming formidable device reliability challenges. Most memristive devices operate on the basis of the conductive filament mechanism. In such systems, three fundamental issues hinder practical applications. First, the stochastic formation and rupture of conductive filaments introduce cycle‐to‐cycle and device‐to‐device variability, leading to poor uniformity. Second, the underlying switching process relies on ionic migration, which inevitably induces microstructural changes in the active layer. Third, the relatively high voltages or currents required for operation not only result in considerable energy consumption but also generate significant Joule heating, further accelerating material degradation. In recent years, researchers have significantly improved the stability of memristors by optimizing device structures and memristive materials. However, fundamentally resolving the stability issues of memristors remains challenging, making it difficult to meet the practical requirements for reliable and stable large‐scale fabrication.

To overcome these limitations, emerging device concepts that eliminate ionic migration offer promising alternatives. Purely electronic memristors represent one such class, in which resistance switching arises from purely electronic phenomena, such as charge trapping and detrapping [160]. The absence of ion movement inherently bypasses microstructural fatigue and stochastic filament dynamics, delivering superior uniformity, endurance, and operational stability. These characteristics render purely electronic memristors highly compatible with the stringent requirements of large‐scale integration. In addition, AOC memristors offer a distinct alternative for addressing the stability limitations of electrically controlled devices [194, 195, 196, 197, 198, 199, 206, 210]. In such devices, conductance modulation is achieved through light‐induced capture and detrapping of electrons at defect sites. This mechanism involves no ionic migration and Joule heating, fundamentally circumventing the microstructural fatigue and thermal degradation of conventional filamentary memristors. The optical writing and erase processes are essentially nondestructive, preserving the stability of the memristive material over extended cycling. As a result, these devices exhibit exceptional stability and reproducibility, providing a compelling pathway toward the reliable large‐scale fabrication required for practical RC hardware.

## 7 Conclusions

In conclusion, the development of memristor‐based RC is poised to move toward more integrated, adaptive, and energy‐efficient neuromorphic computing platforms. Future systems may leverage the intrinsic dynamics and multi‐functionality of advanced memristive materials to enable real‐time processing of heterogeneous sensory data with minimal hardware footprint. Such systems hold significant promise for applications in edge computing, robotic sensing, and brain‐inspired artificial intelligence, where low‐power, high‐speed, and robust multimodal signal analysis is essential. Continued exploration of novel device mechanisms and system‐level integration will further expand the applicability and performance of memristive RC in next‐generation intelligent systems.

## Conflicts of Interest

The authors declare no conflicts of interest.

## Data Availability

The data that support the findings of this study are available from the corresponding author upon reasonable request.
